# MCT1‐mediated Lactate Shuttle to Mitochondria Governs Macrophage Polarization and Modulates Glucose Homeostasis by Affecting β Cells

**DOI:** 10.1002/advs.202414760

**Published:** 2025-07-14

**Authors:** Lingling Chen, Yijun Lin, Xinyu Zhu, Shixuan Zhuo, Zixuan Li, Cheng Guo, Xiaoyi Ye, Jinzhu Chen, Shuying Wang, Yan Chen

**Affiliations:** ^1^ CAS Key Laboratory of Nutrition Metabolism and Food Safety Shanghai Institute of Nutrition and Health University of Chinese Academy of Sciences Chinese Academy of Sciences Shanghai 200031 China; ^2^ Xiamen Cardiovascular Hospital School of Medicine Xiamen University Xiamen 361016 China; ^3^ School of Life Science and Technology ShanghaiTech University Shanghai 201210 China

**Keywords:** glucose homeostasis, lactate shuttle, macrophage, MCT1, mitochondria, pancreatic islet

## Abstract

As a major fuel source for the tricarboxylic acid cycle, lactate controls energy metabolism through cell‐to‐cell or tissue‐to‐tissue lactate shuttles via monocarboxylate transporters (MCTs). Although lactate is shown to influence macrophage functions via histone lactylation, the specific functions of MCTs in macrophages remain incompletely understood. This study discovers that MCT1 and MCT4 have contrasting effects on regulating macrophage polarization. M1 polarization is associated with increase of MCT4 while M2 polarization is accompanied with increase of MCT1. MCT1 is mainly localized in mitochondria while MCT4 is localized on the plasma membrane. M1 polarization elevates lactate efflux from the cytoplasm to extracellular space, while M2 polarization increases intracellular lactate flux to mitochondria. At cellular level, blocking MCT1 exacerbates LPS‐induced M1‐like polarization and impairs mitochondria function. At animal level, deletion of MCT1 in macrophages exacerbates glucose intolerance, suppresses insulin secretion and increases islet cell death in high‐fat diet fed mice. Mechanistically, lactate reduces insulin secretion through GPR81‐cAMP‐PKA signaling pathway. These findings not only disclose that the MCT1‐mediated intracellular lactate shuttle to the mitochondria plays a pivotal role in governing macrophage polarization but also uncovers a functional interplay between macrophages and β cells in maintaining glucose homeostasis.

## Graphical abstract

This study demonstrates that the transport of lactate to the mitochondria is of critical importance in determining the functions of macrophages by altering mitochondrial respiration. In macrophages, this lactate transport mechanism regulates glucose homeostasis by influencing the functions of insulin‐secreting cells in the pancreatic islets. As such, it establishes a functional connection between lactate and diabetes.

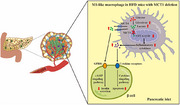

## 1 Introduction

Macrophages can be polarized into a classically activated state, termed “M1‐like,” by lipopolysaccharide (LPS) or interferon‐γ (IFN‐γ), or into an alternative anti‐inflammatory state, called “M2‐like,” by interleukin‐4 (IL‐4). These two macrophage subtypes exhibit distinct metabolic characteristic^[^ ^1^ ^]^ M1‐like subtype is characterized by high glycolysis and low mitochondrial activity,^[^ ^2^, ^3^, ^4^ ^]^ while M2‐like subtype is high in mitochondrial oxidation and glutamine metabolism.^[^ ^5^, ^6^ ^]^ Metabolic reprogramming in macrophages is of critical importance as it supplies the energy sources and metabolites necessary for sustaining the diverse functions of these macrophages. For example, the decreased activity of isocitrate dehydrogenase (IDH) in the TCA cycle leads to an increase of itaconate that enhances the inflammatory activity of M1‐like macrophages,^[^ ^4^ ^]^ while a reduction in succinate dehydrogenase (SDH) activity in the TCA cycle is responsible for the elevation of succinate that stimulates the production of pro‐inflammatory cytokines via HIF‐1α.^[^ ^3^ ^]^ In M2‐like macrophages, increases in glutamine metabolism and fatty acid oxidation fuel th e TCA cycle to maintain high rate of mitochondrial oxidation.^[^ ^7^, ^8^ ^]^ These observations highlight that metabolic reprogramming is a crucial feature of macrophage polarization. However, how lactate flux regulates metabolic reprogramming in macrophages during the polarization process remains elusive. This constituted the key question that our study endeavored to answer.

Lactate produced by glycolysis used to be considered as a metabolic “waste.” However, accumulating evidence in recent years has revealed the important functions of lactate in regulating metabolic homeostasis.^[^ ^9^, ^10^ ^]^ The lactate shuttle theory depicts the active roles of lactate in tissue‐to‐tissue and cell‐to‐cell communications between lactate‐producing cells and lactate‐consuming cells.^[^ ^11^ ^]^ This theory is supported by a recent discovery demonstrating that lactate serves as a major fuel to feed the TCA cycle,^[^ ^12^ ^]^ while lactate homeostasis itself is maintained through its regulation on glycolysis and lipolysis.^[^ ^13^ ^]^ Lactate can modulate the functions of multiple proteins via lactylation. It was found that lactate regulates macrophage metabolism via epigenetic modulation through lactylation of histone.^[^ ^14^ ^]^ Recently, alanyl‐tRNA synthases (AARS1 and AARS2) were discovered to be lactate sensors and a lactyltransferase to mediate lactylation of proteins.^[^ ^15^, ^16^, ^17^ ^]^ Lactate may also function as signaling molecule to regulate energy metabolism through modulating lipolysis of adipose tissues via G‐protein coupled receptor GRP81.^[^ ^18^ ^]^

It has long been recognized that, compared to non‐diabetic individuals, patients with diabetes, including both type 1 and type 2 diabetes, have elevated blood lactate levels.^[^ ^19^, ^20^, ^21^ ^]^ A study demonstrated that the plasma lactate concentration is highest in obese subjects with type 2 diabetes when compared to non‐obese and non‐diabetic individuals.^[^ ^20^ ^]^ Moreover, individuals with an impaired glucose tolerance test also had significantly higher blood lactate levels than those with normal glucose tolerance.^[^ ^22^ ^]^ Previous studies have attempted to elucidate how metabolic alterations contribute to the elevated blood lactate levels in diabetic patients. For instance, it was suggested that the elevation of blood lactate is linked to an enhancement in carbohydrate oxidation in type 2 diabetes.^[^ ^23^ ^]^ Additionally, the proposed mechanisms underlying diabetes‐associated hyperlactatemia involve changes in intracellular glucose metabolism within insulin‐sensitive tissues. These changes include reduced glycogen synthesis, impaired glucose oxidative metabolism, and an increased whole‐body rate of non‐oxidative glycolysis.^[^ ^24^, ^25^, ^26^ ^]^ However, it remains unclear whether the increased blood lactate concentration plays a causal role in the pathogenesis of diabetes. This is an issue that will be investigated in the present study.

Lactate traverses the plasma membrane via monocarboxylate transporters 1–4 (MCT1 to MCT4, encoded by *Slc16a1*, *Slc16a7*, *Slc16a8*, and *Slc16a3*, respectively).^[^ ^27^ ^]^ It has been discovered that, in a high‐lactate microenvironment, MCT4, rather than MCT1, is accountable for the efflux of lactate in macrophages and other cells.^[^ ^28^ ^]^ The functions of MCT1 in regulating metabolic homeostasis have been recognized in recent years. MCT1 in adipocytes can regulate systemic insulin resistance via modulating apoptosis of adipocytes and inflammation of adipose tissue in obese mice.^[^ ^29^ ^]^ MCT1 in the liver controls hepatic steatosis vis regulating PPARα.^[^ ^30^ ^]^ MCT1 in the intestinal epithelium modulates systemic inflammation and insulin sensitivity in a sex‐dimorphic manner.^[^ ^31^ ^]^ MCT1 in skeletal muscle can affect muscle fiber switching through regulating TCA flux.^[^ ^32^ ^]^ A recent study revealed that lactate secreted by endothelial cells can regulate muscle regeneration via affecting M2 polarization of macrophage via MCT1.^[^ ^33^ ^]^ However, the functions of MCT1 in macrophages, especially its involvement in regulating metabolic homeostasis, remain incompletely characterized.

In this study, we comprehensively analyzed the functions of MCT1 in macrophage polarization primarily through the examination of a mouse model with MCT1 deletion in macrophages. We determined that MCT1 and MCT4 have opposing effects on the regulation of macrophage polarization. Significantly, we uncovered that M1 polarization elevates lactate efflux from the cytoplasm to extracellular space, while M2 polarization increases intracellular lactate flux to mitochondria. Additionally, we found that MCT1 in macrophages can influence glucose homeostasis by regulating insulin secretion through the GPR81‐cAMP‐PKA signaling pathway. Therefore, our study not only reveals the function of MCT1 and MCT4 in macrophage polarization but also uncovers a functional connection between lactate and the pathogenesis of diabetes.

## 2 Results

### 2.1 MCT1 and MCT4 have Opposite Effects on Macrophage Polarization

**Figure 1:**
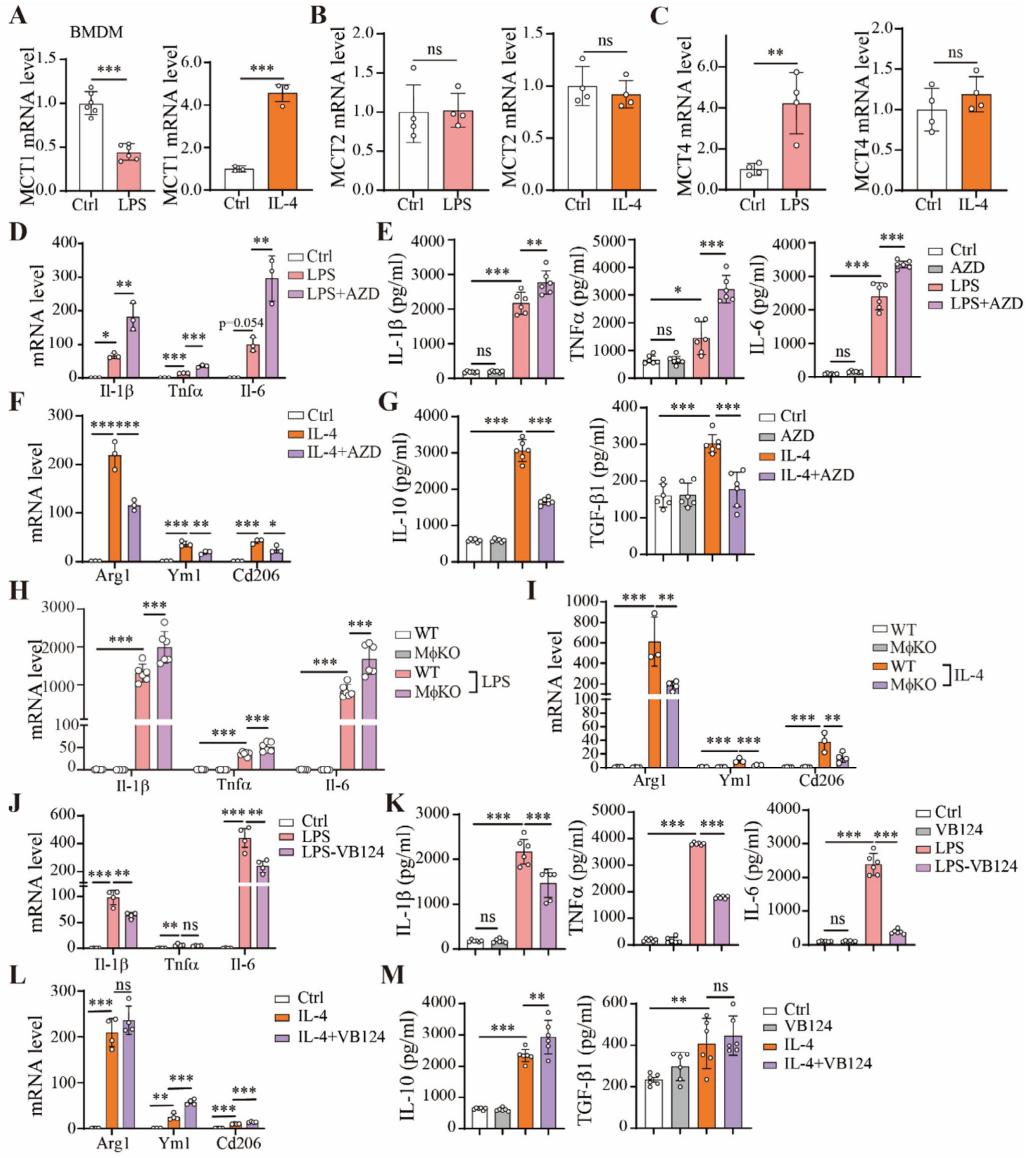
Distinct functions of MCT1 and MCT4 in macrophage polarization. A–C) The mRNA levels of genes encoding MCT1, MCT2 and MCT4 in BMDMs from C57BL/6J mice treated with LPS (100 ng mL^−1^) for 12 h or IL‐4 (50 ng mL^−1^) for 24 h. n = 6 for LPS‐treated group and n = 3 for IL‐4‐treated group in A, n = 4 per group in B and C. The same concentrations of LPS and IL‐4 were used in cell treatment throughout this article. D) The mRNA levels of *Il‐1β*, *Tnfα* and *Il‐6* in BMDMs treated with LPS in the presence or absence of 100 nM AZD3965 (this concentration is used in cell treatment covered in this article) for 12 h, n = 3 per group. E) IL‐1β, TNFα and IL‐6 concentrations in the culture medium of BMDMs as in D, n = 6 per group. F) The mRNA levels of *Arg1*, *Ym1*, and *Cd206* in BMDMs treated with IL‐4 in the presence or absence of AZD3965 for 24 h, n = 3 per group. G) IL‐10 and TGF‐β1 concentrations in the culture medium of BMDMs as in F, n = 6 per group. H) The mRNA levels of pro‐inflammatory genes in BMDMs isolated from *Slc16a1* ^f/f^ (WT) and *Slc16a1* ^f/f^ Lyz2^cre/‐^ (MφKO) mice, treated with LPS for 12 h, n = 6 per group. I) The mRNA levels of anti‐inflammatory genes in BMDMs as in H but treated with IL‐4 for 24 h, n = 3 for WT+IL‐4 group, n = 4 for other groups. J) The mRNA levels of pro‐inflammatory genes in BMDMs treated with LPS in the presence or absence of 10 µM VB124 for 12 h, n = 4 per group. K) IL‐1β, TNFα and IL‐6 concentration in culture medium of BMDMs as in J, n = 6 per group. L) The mRNA levels of anti‐inflammatory genes in BMDMs treated with IL‐4 in the presence or absence of 10 µM VB124 for 24 h, n = 4 per group. M) IL‐10 and TGF‐β1 concentrations in culture medium of BMDMs as in L, n = 6 per group. Data are presented as mean ± SD with each point representing a biological replicate and n representing the number of biological replicates. Data are representative of three independent experiments (A, D, F, H and I) and two independent experiments (B, C, E, G and J–M). The *p* values were calculated using unpaired, two‐sided Student's *t*‐test (A–C) or one‐way ANOVA with Tukey's honest significant difference (HSD) pose hoc analysis (D–M). *p < 0.05, **p < 0.01, ***p < 0.001, ns for non‐significant. AZD, AZD3965.

The major lactate transporters in macrophages are MCT1, MCT2 and MCT4.^[^ ^34^ ^]^ At first, we analyzed whether expression level of these three MCTs were altered by polarization in macrophages. When bone marrow‐derived macrophages (BMDMs) were induced by LPS to polarize into a classically‐activated state (M1‐like), the MCT1 mRNA level was significantly reduced while the MCT4 mRNA level was significantly increased (**Figure** **1**A,C), consistent with a previous report.^[^ ^35^ ^]^ In contrast, when BMDMs were polarized to an alternatively‐activated state (M2‐like) by IL‐4 treatment, MCT1 expression was markedly elevated (Figure 1A). On the other hand, the mRNA level of MCT2 was not altered by macrophage polarization (Figure 1B). These data thus indicated that MCT1 and MCT4, but not MCT2, are likely involved in macrophage polarization. We next investigated how MCT1 affects macrophage polarization. Inhibition of MCT1 by a specific inhibitor AZD3965 in BMDMs was able to enhance LPS‐induced expression of pro‐inflammatory cytokines IL‐1β, TNFα and IL‐6 (Figure 1D), while increasing secretion of these pro‐inflammatory cytokines in the culture medium (Figure 1E). Consistently, LPS‐induced expressions of IL‐1β, TNFα and IL‐6 were all enhanced by knockdown of *Slc16a1* in RAW264.7 cells (Figure S1, Supporting Information). However, IL‐4‐induced expression of M2 macrophage markers including Arg1, Ym1 and Cd206 was reduced by AZD3965 treatment in BMDMs (Figure 1F), while IL‐4‐induced secretion of IL‐10 and TGF‐β1 was suppressed by AZD3965 (Figure 1G).

To further explore the functions of MCT1 in macrophages, we deleted the *Slc16a1* gene in macrophages by crossing *Slc16a1* ^f/f^ mice (named as WT) with Lyz2‐Cre mice to generate *Slc16a1* ^f/f^‐Lyz2^cre/‐^ mice (named as MφKO) (Figure S2A, Supporting Information). Successful deletion of *Slc16a1* gene was confirmed by analyzing MCT1 expression through both RT‐PCR and Western blotting (Figure S2B,C, Supporting Information). Consistently, we found that deletion of *Slc16a1* enhanced LPS‐induced expression of IL‐1β, TNFα and IL‐6 in BMDMs (Figure 1H), while IL‐4‐induced expression of M2 macrophage markers was reduced by *Slc16a1* deletion (Figure 1I).

We also analyzed the effect of *Slc16a1* deletion on LPS‐induced inflammation in vivo. In the LPS‐induced acute peritonitis mouse model, the percentage of F4/80+CD11b+ macrophages were significantly increased in the blood of MφKO mice (Figure S3A, Supporting Information). Macrophage infiltration in the liver was increased by *Slc16a1* deletion (Figure S3B, Supporting Information). The blood levels of IL‐1β, TNFα and IL‐6 were also increased by *Slc16a1* deletion (Figure S3C, Supporting Information). These results further indicated that MCT1 has a negative effect on M1‐like polarization of macrophages.

In addition to MCT1, MCT4 was reported to be another major type of lactate transporter in the macrophages.^[^ ^36^ ^]^ Treatment of BMDMs with LPS led to upregulation of MCT4 mRNA (Figure 1C), opposite to the finding with MCT1 (Figure 1A). We analyzed whether macrophage polarization was altered by blocking MCT4 by a specific inhibitor VB124.^[^ ^37^ ^]^ VB124 was able to reduce LPS‐induced expression and secretion of IL‐1β, TNFα and IL‐6 (Figure 1J,K), while enhancing some of the M2‐like macrophage markers (Figure 1L) and secretion of IL‐10 (Figure 1M), consistent to a previous observation.^[^ ^36^ ^]^ Therefore, MCT4 has a positive effect on M1‐like polarization and a negative effect on M2‐like polarization, indicating that MCT1 and MCT4 have opposite effects on regulating macrophage polarization.

We also analyzed whether blocking MCT1 and MCT4 altered the functions of macrophages. Blocking MCT1 with AZD3965 increased phagocytosis and migration of BMDMs in the absence and presence of LPS (Figure S4, Supporting Information). Blocking MCT4 with VB124 reduced phagocytosis and migration of BMDMs in the absence and presence of LPS (Figure S4, Supporting Information). IL‐4 treatment itself reduced phagocytosis and migration of macrophages, and such reduction was cancelled out by AZD3965 treatment (Figure S4, Supporting Information). These results, therefore, indicated that MCT1 and MCT4 can affect the functions of macrophages.

### 2.2 MCT1 Affects Mitochondrial Respiration of Macrophages

**Figure 2:**
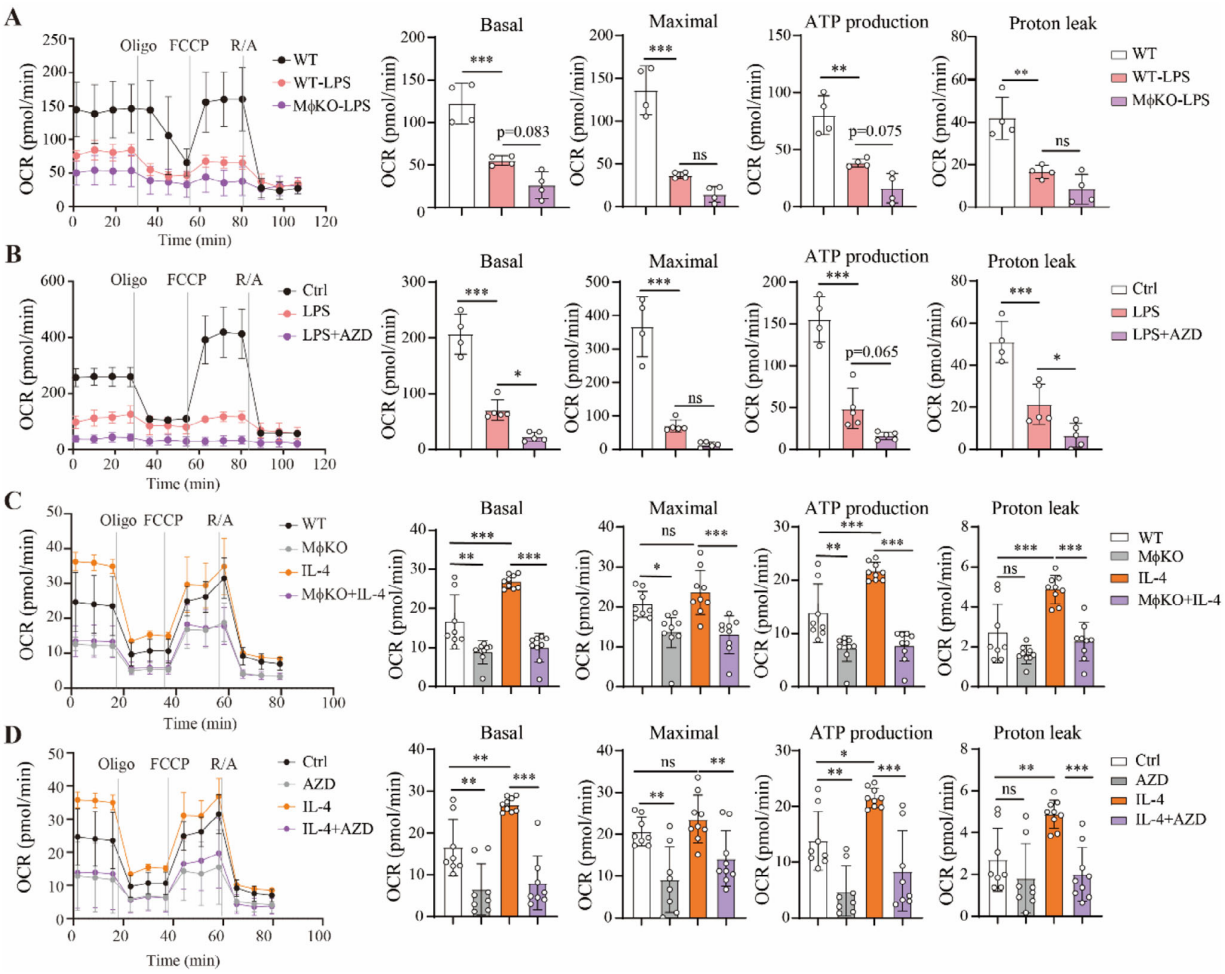
Deletion or inhibition of MCT1 in macrophages causes decline of mitochondrial respiration. A) OCR of BMDMs from WT and MφKO mice measured using Seahorse Bioanalyzer after treatment with or without LPS for 12 h, n = 4 per group,10^5^ cells per group. B) OCR of BMDMs treated with LPS and AZD3965 for 12 h, n = 4 for control group, n = 5 for the other two groups, 10^5^ cells per group. C) OCR of BMDMs treated with IL‐4 for 24 h, n = 8 for WT group, n = 9 for the other three groups, 10^4^ cells per group. D) OCR of BMDMs treated with IL‐4 and AZD3965 for 24 h, n = 8 for WT and AZD‐treated group, n = 9 for IL‐4 and IL‐4+AZD group, 10^4^ cells per group. Data are presented as mean ± SD with each point representing a biological replicate and n representing the number of biological replicates. Data are representative of three independent experiments(A, B) and representative of two independent experiments (C, D). The *p* values were calculated using one‐way ANOVA with Tukey's honest significant difference (HSD) pose hoc analysis. *p < 0.05, **p < 0.01, ***p < 0.001, ns for non‐significant. AZD, AZD3965.

As lactate is a major fuel for feeding the TCA cycle,^[^ ^12^ ^]^ we next analyzed the function of MCT1 on mitochondria energy metabolism of macrophages. Loss of MCT1 led to a significantly attenuated mitochondrial activity in BMDMs under LPS stimulation, shown as decreases in basal respiration, maximal respiration, ATP production and proton leak (**Figure** **2**A). Similar observations were also found in BMDMs treated with MCT1 inhibitor AZD3965 (Figure 2B). These results thus demonstrated that MCT1 loss would impair mitochondrial respiration, likely contributing to enhancement of LPS‐induced pro‐inflammatory activity of macrophages.

Subsequently, we investigated the impact of MCT1 on mitochondrial activity in macrophages undergoing IL‐4‐induced M2‐like polarization. Under basal conditions, the absence of MCT1 led to a reduction in mitochondrial respiration, as shown in Figure 2C. Significantly, deletion of MCT1 completely eliminated the stimulatory effect of IL‐4 on mitochondrial respiration. The same outcome was replicated when using AZD3965. Specifically, AZD3965 entirely abolished the IL‐4‐induced enhancement of mitochondrial respiration (Figure 2D). Collectively, these data demonstrate that MCT1 is essential for regulating mitochondrial functions. These findings, therefore, further supported our conclusion that MCT1 plays a pivotal role in governing macrophage polarization.

### 2.3 MCT1 Mediates Intracellular Lactate Shuttle from Cytoplasm to Mitochondria in a Manner that Depends on Macrophage Polarization

**Figure 3:**
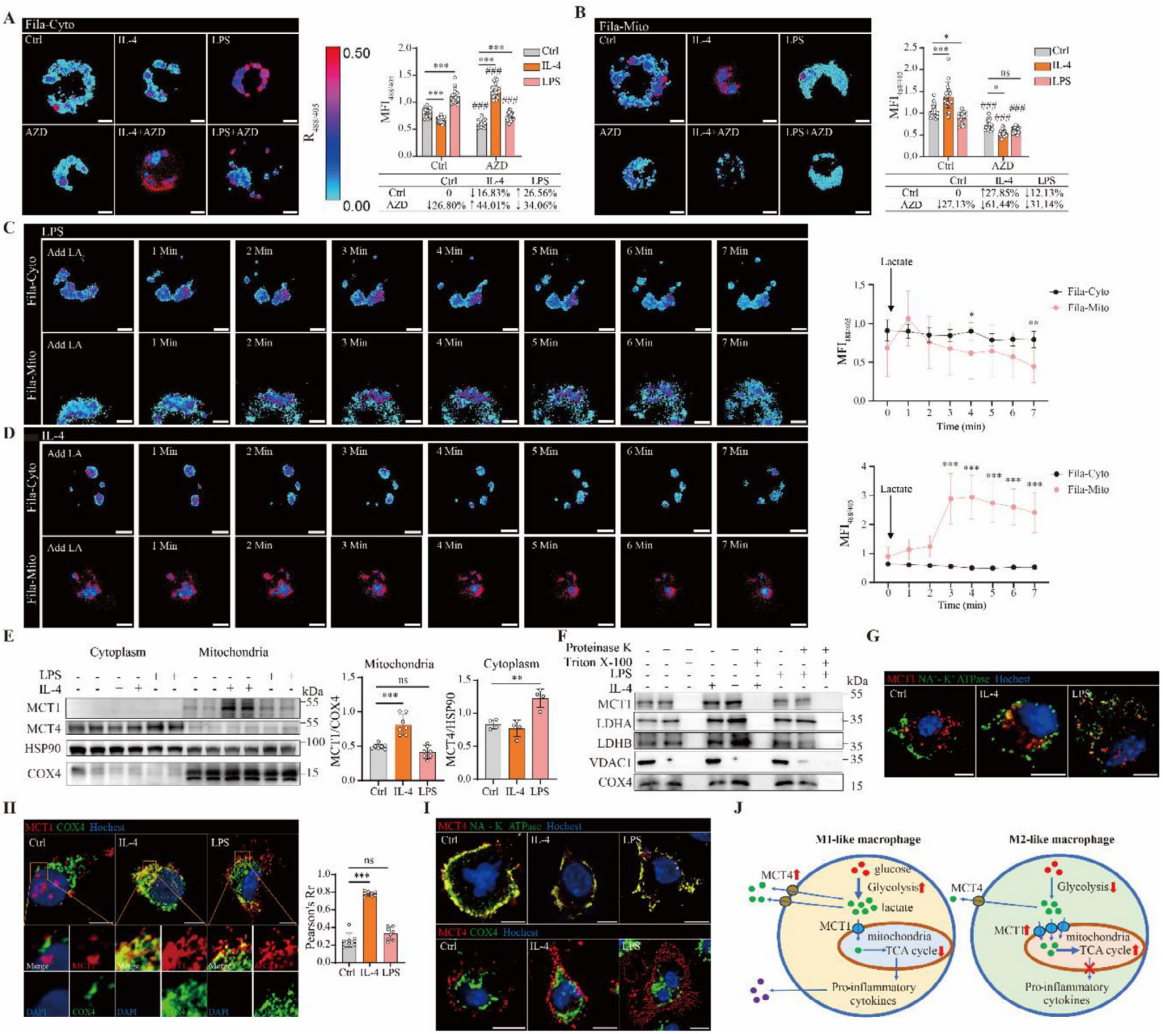
Blocking MCT1 reduces lactate transport to the mitochondria. A, B) Ratiometric fluorescence images of RAW264.7 cells expressing lactate tracers Fila‐Cyto and Fila‐Mito that measures lactate level in cytosol and mitochondria, respectively. Images were pseudo‐colored by R488/405. Cells were treated with LPS or IL4 in the presence or absence of AZD3965 for 12 h. Scale bar, 5 µm. Quantification result of the ratio of mean immunofluorescence intensity (MFI) 488 to 405 is shown on the right, n = 15–20 for each group. C, D) Kinetics of Fila fluorescence in RAW264.7 cells in response to 10 mM lactate after treatment with LPS or IL‐4 for 12 h. Images were pseudo‐colored by R488/405. Quantification result of MFI 488 to 405 is on the right, n = 11 for Fila‐Cyto and n = 9 for Fila‐Mito in C, n = 10 for Fila‐Cyto and n = 7 for Fila‐Mito in D. E) Western blotting analysis of MCT1 and MCT4 in cytoplasmic and mitochondrial fractions from BMDMs upon treatment with LPS or IL‐4 for 12 h. Quantitation results of MCT4/HSP90 and MCT1/COX4 are shown in the right panel. F) Western blotting analysis of mitochondria compartment isolated from BMDMs treated with LPS or IL‐4 and then treated with proteinase K (50 µg mL^−1^) in the presence or absence of 1% Triton X‐100. G–I) Representative images of MCT1, MCT4, NA^+^‐K^+^ ATPase, and COX4 immuno‐fluorescence staining of RAW264.7 cells treated with LPS or IL‐4 for 12 h, respectively. Pearson's correlation coefficient of the localization of MCT1 with COX4 is shown on the right for H. n = 8. J) A diagram to depict the roles of MCT1 and MCT4 in macrophage polarization. Data are presented as mean ± SD with each point representing a biological replicate and n representing the number of biological replicates. Data are representative of three independent experiments (A, B, E‐G, I) and representative of two independent experiments (C, D, F). The *p* values were calculated using two‐way ANOVA with Tukey's honest significant difference (HSD) pose hoc analysis (A, B) or one‐way ANOVA with Tukey's HSD pose hoc analysis (C–E, H). # represents the *p* value between corresponding groups with and without AZD3965 treatment (A, B). * indicates the statistical significance between each time point of Fila‐Cyto group compared to Fila‐Mito group (C and D). *p < 0.05, **p < 0.01, *** and ###*p* < 0.001, ns for non‐significant. AZD, AZD3965. Scale bar: 5 µm.

We next explored the molecular mechanism underlying the inhibition of mitochondrial respiration by loss of MCT1. The lactate shuttle theory illustrates two types of lactate shuttles: cell‐to‐cell shuttle (from lactate‐producing cells to lactate‐consuming cells) and intracellular shuttle (from cytoplasm to mitochondria) as proposed by Brooks.^[^ ^11^ ^]^ We hypothesized that MCT1 participates in the intracellular lactate shuttle within macrophages. As a result, the absence of MCT1 would impede lactate transport to the mitochondria, thereby causing a decline in mitochondrial respiration. To test this hypothesis, we first analyzed the lactate levels in both the cytoplasm and mitochondria of macrophages using newly developed fluorescent lactate tracers,^[^ ^38^ ^]^ in which Fila‐Cyto measures the cytoplasmic lactate level while Fila‐Mito reports the mitochondrial lactate level. LPS treatment increased the cytoplasmic lactate level by 26.6% while reducing the mitochondrial lactate level by 12.1% in RAW264.7 cells (**Figure** **3**A,B). In contrast, IL‐4 treatment reduced the cytoplasmic lactate level by 16.8% while increasing the mitochondrial lactate level by 27.9% in RAW264.7 cells (Figure 3A,B). These results demonstrated that M1‐like polarization is associated with a decreased flux of lactate from the cytoplasm to the mitochondria, whereas M2‐like polarization shows an increased flux in this regard. Most importantly, inhibition of MCT1 function by AZD3965 reduced the mitochondrial lactate level under basal condition and upon treatment with LPS or IL‐4 (Figure 3B), indicating that MCT1 can mediate the transport of lactate from cytoplasm to mitochondria. It was also notablely that under the condition of LPS treatment, inhibition of MCT1 further reduced the mitochondrial lactate level (Figure 3B). Under the condition of IL‐4 treatment, inhibition of MCT1 increased the cytoplasmic lactate level while reducing the mitochondrial lactate level (Figure 3A,B). These observations, therefore, might underlie our findings that loss of MCT1 promotes M1‐like polarization while inhibiting M2‐like polarization of macrophages as found in Figure 1.

Next, we investigated the time‐course of lactate distribution in cytoplasm and mitochondria upon lactate treatment in macrophages. Under LPS treatment, administration of lactate led to a slow increase in the cytoplasmic lactate level within 7 min, while resulting in a rapid raise of the mitochondrial lactate level in 1 min, followed by gradual decline (Figure 3C). Under IL‐4 treatment, administration of lactate led to a robust increase in the mitochondrial lactate level, while causing minimal change in the cytoplasmic lactate level (Figure 3D). These results further support our hypothesis that M1‐like polarization reduces the flux of lactate from cytoplasm to mitochondria, while M2‐polarization enhances this flux.

Consistent with our observations, we found that MCT1 protein level in the mitochondria fraction of BMDMs was markedly increased by IL‐4 treatment, while MCT4 protein level in the cytoplasmic fraction was elevated by LPS treatment (Figure 3E and Figure S5, Supporting Information). To further elucidate the localization of MCT1 on the mitochondria, we conducted a proteinase K experiment using mitochondria isolated from BMDMs. In this experiment, we utilized VDAC1 as a marker for the outer mitochondrial membrane and COX4 as a marker for the inner mitochondrial membrane. Proteinase K treatment led to the degradation of VDAC1, while MCT1 and COX4 remained intact in the absence of Triton X‐100 (Figure 3F). However, in the presence of Triton X‐100, proteinase K treatment degraded all three proteins (Figure 3F). In addition, we found that LDHA and LDHB were also present in the inner mitochondrial membrane. These results not only demonstrate that MCT1 is localized on the inner mitochondrial membrane but also indicate that the level of mitochondria‐localized MCT1 is increased in M2‐like macrophages.

Our conclusion was further supported by immunofluorescent staining studies in RAW264.7 cells. MCT1 had no apparent localization on the plasma membrane (Figure 3G), while its mitochondrial localization was markedly elevated by IL‐4 treatment (Figure 3H and Figure S6A, Supporting Information). In contrast, MCT4 was only found on the plasma membrane, and IL‐4 treatment could not increase mitochondrial localization of MCT4 (Figure 3I and Figure S6B, Supporting Information).

In CD8(+) T cells, it has been discovered that lithium can divert lactate to the mitochondria and this occurs by enhancing the mitochondrial localization of MCT1 via the diacylglycerol‐PKCθ signaling pathway.^[^ ^39^ ^]^ To determine whether PKCθ signaling is essential for the IL‐4‐enhanced mitochondrial localization of MCT1, we evaluated the impact of the PKCθ inhibitor sotrastaurin (STN) in BMDMs.^[^ ^40^ ^]^ Our findings revealed that STN not only decreased the IL‐4‐augmented mitochondrial localization of MCT1 but also diminished the IL‐4‐induced secretion of IL‐10 (Figure S7A, Supporting Information). Surprisingly, we found that STN treatment increased nuclear localization of MCT1 (Figure 6A), a phenomenon that has not been reported in literature and is worthy future investigation. Western blotting analysis also confirmed that IL‐4‐stimulated mitochondrial localization of MCT1 was diminished by STN treatment (Figure S7B, Supporting Information), while IL‐4‐induced IL‐10 production was reduced by STN treatment (Figure S7C, Supporting Information). Collectively, these results thus suggest that PKCθ signaling is likely implicated in the IL‐4‐mediated mitochondrial localization of MCT1 and M2 polarization of macrophages.

In theory, both pyruvate and lactate can act as fuels for the TCA cycle, being transported via MPC and MCT1, respectively. We investigated the effects of blocking MPC with UK5099^[^ ^41^ ^]^ and blocking MCT1 with AZD3965 in BMDMs. We performed a preliminary experiment to analyze the metabolic flux of extracellularly added U‐^13^C‐glucose. In LPS‐induced M1‐like macrophages derived from BMDMs, AZD3965 treatment significantly reduced most of the M+2 and M+4 forms of TCA intermediates (Figure S8, Supporting Information), consistent with our observation that blocking MCT1 inhibits TCA cycle. Surprisingly, UK5099 had no apparent effect to decrease the M+2 and M+4 forms of TCA intermediates (Figure S8, Supporting Information), suggesting pyruvate through MPC might not be a major fuel source of TCA cycle in these macrophages. These results thus imply that lactate imported by MCT1, rather than pyruvate imported by MPC, serves as the primary fuel for the TCA cycle in the mitochondria of macrophages. This is an interesting issue that merits in‐depth exploration in future studies.

Based on our findings, we present a model to illustrate the functional contributions of MCT1 and MCT4 in macrophage polarization (Figure 3J). We propose that MCT4 is predominantly located in the plasma membrane and facilitates efflux of lactate to the extracellular space. Conversely, MCT1 is mainly situated on the mitochondrial membrane, mediating the intracellular lactate shuttle by transporting lactate from the cytoplasm into the mitochondria. In M1‐like macrophages, the low mitochondrial localization of MCT1 restricts lactate transport to the mitochondria. This, combined with enhanced glycolysis,^[^ ^1^ ^]^ results in an accumulation of cytoplasmic lactate. As a consequence, M1‐like macrophages exhibit increased efflux of lactate to the extracellular space via MCT4. On the contrary, during M2 polarization of macrophages, the mitochondrial localization of MCT1 is upregulated. This leads to augmented lactate transport into the mitochondria, thereby enhancing the TCA cycle. We further propose that the variance in lactate transport to the mitochondria via MCT1 is a key factor in the modulation of mitochondrial function during macrophage polarization, given that lactate serves as a fuel source for the TCA cycle. In essence, the MCT1‐mediated intracellular lactate shuttle is pivotal in the metabolic reprogramming that occurs during macrophage polarization.

### 2.4 Slc16a1 Deletion in Macrophages Aggravates Insulin Resistance, Reduces Insulin Secretion and Increases Systemic Inflammation in HFD Mice

**Figure 4:**
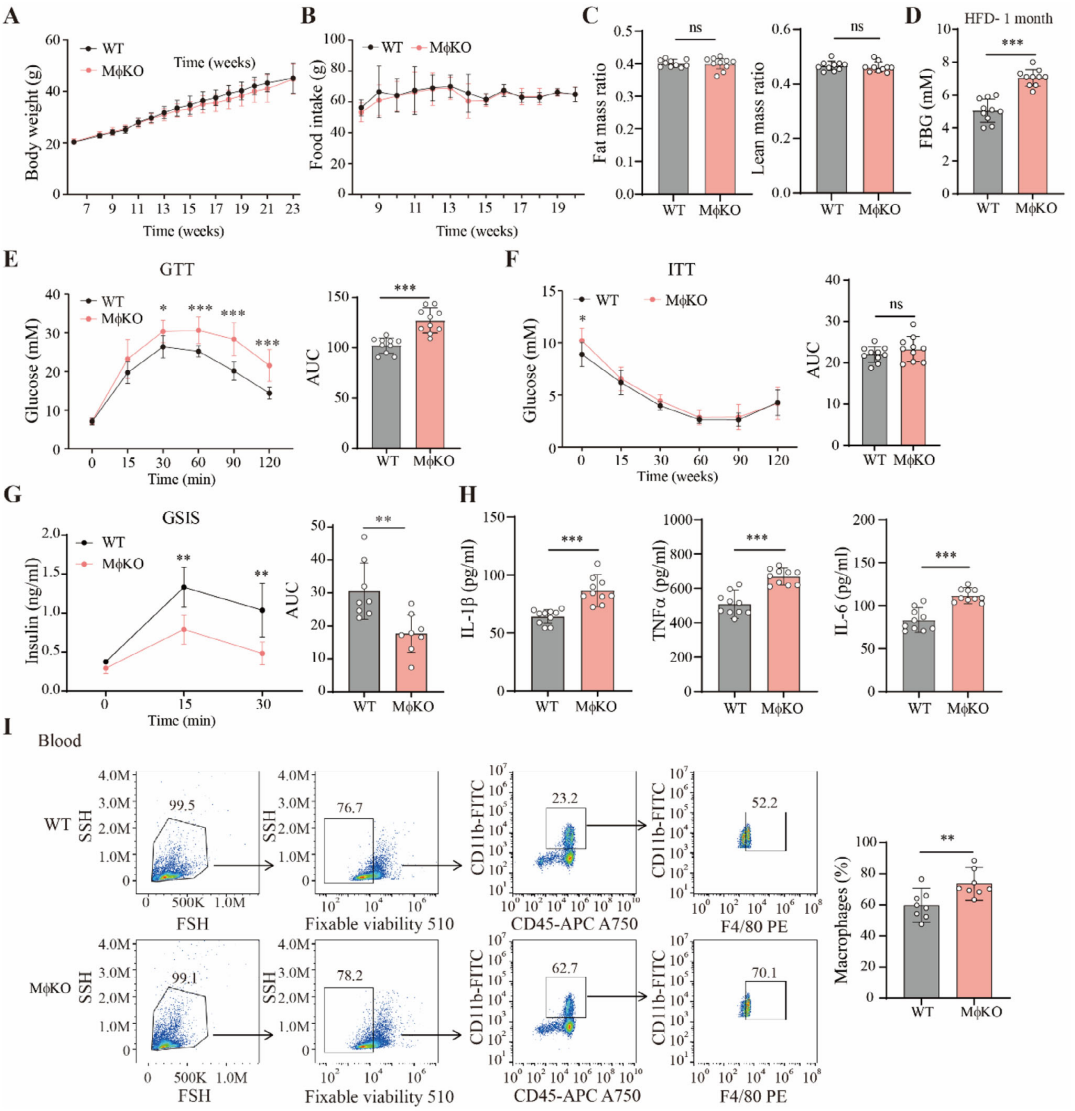
MCT1 deletion in macrophage aggravates glucose intolerance, inhibits insulin secretion and promotes systemic inflammation in HFD mice. A) Body weight of WT and MφKO male mice fed with HFD for 16 weeks. HFD was started at 7 week‐age, n = 10 mice per group. B) Food intake of WT and MφKO mice as in A, n = 10 mice per group. C) Rate of fat mass and lean mass in WT and MφKO mice at the endpoint, n = 10 mice per group. D) Fasting blood glucose (FBG) of WT and MφKO mice at 1 month after HFD. The mice were fasted for 16 h before the test, n = 10 mice per group. E) Glucose tolerance test (GTT) of the mice at 12th week after HFD. The mice were fasted for 16 h before the test, n = 10 mice per group. Quantification of area under curve (AUC) is shown on the right. F) Insulin tolerance test (ITT) of the mice at 13th week after HFD, n = 10 mice per group. AUC is shown on the right. G) Glucose stimulated insulin secretion (GSIS) of the mice at 14th week after HFD, n = 8 mice per group. AUC is shown on the right. H) IL‐1β, TNFα and IL‐6 concentrations in the mouse serum at the endpoint, n = 10 mice per group. I) Representative flow cytometry plot and ratio of V510^‐^&CD45^+^CD11b^+^&F4/80^+^ macrophages in the blood of the mice as in A, n = 8 mice per group. Data are presented as mean ± SD. Data are representative of two independent experiments. The *p* values were calculated using one‐way ANOVA with Tukey's honest significant difference (HSD) pose hoc analysis (A, B, E–G) or unpaired, two‐sided Student's *t*‐test (C, D, H, I and AUC analysis of E‐G). *p < 0.05, **p < 0.01, ***p < 0.001, ns for non‐significant. AZD, AZD3965.

Next, we investigated the in vivo functions of MCT1 loss in macrophages. We fed 7‐week‐aged WT and MφKO mice with a high‐fat diet (HFD) for 16 weeks. Intriguingly, we found that MCT1 loss in macrophages did not impact body weight, food intake and the ratio of fat and lean mass (**Figure** **4**A–C). However, the fasting blood‐glucose (FBG) was dramatically increased in MφKO mice after HFD feeding (Figure 4D). Next, we analyzed glucose tolerance (GTT) of the mice. MCT1 deficiency in macrophages aggravated HFD‐induced glucose tolerance (Figure 4E). However, insulin sensitivity of the mice, as measured by insulin tolerance test (ITT), was not altered by *Slc16a1* deletion (Figure 4F). However, the glucose‐stimulated insulin secretion test (GSIS) was impaired in MφKO mice (Figure 4G).

We next analyzed the inflammation status of the mice. The blood levels of IL‐1β, TNFα and IL‐6 were all elevated in MφKO mice (Figure 4H). Similarly, we observed that the CD45^+^CD11b^+^ & F4/80^+^ macrophages were significantly increased in the blood of MφKO mice, as analyzed by flow cytometry (Figure 4I). These results thus indicate that *Slc16a1* deletion in macrophages aggravates glucose intolerance, reduces insulin secretion and increases systemic inflammation in HFD mice, while it has no effect on HFD‐induced obesity.

We also analyzed the mice under normal chow diet. Deletion of MCT1 in macrophages did not alter body weight and the ratio of fat and lean mass (Figure S9A,B, Supporting Information). Deletion of *Slc16a1* had no effect on oral glucose tolerance test (OGTT) (Figure S9C, Supporting Information). We observed no change in insulin secretion in MφKO mice (Figure S9D, Supporting Information). In addition, insulin secretion with isolated islets was not altered by *Slc16a1* deletion (Figure S9E, Supporting Information). These results indicate that β cell function remains intact in normal chow‐fed mice with MCT1 deficiency in macrophages. Since glucose homeostasis is disrupted only in MφKO mice fed HFD, which is known to induce chronic inflammation in various tissues including pancreatic islets, we hypothesized that MCT1 in macrophages influences islet functions solely under inflammatory conditions. Based on these results, we concluded that β cell development per se is unlikely to be affected in MφKO mice.

### 2.5 MCT1 Loss Aggravates Islet Damage and Increases Macrophage Infiltration in Islets of HFD Mice

**Figure 5:**
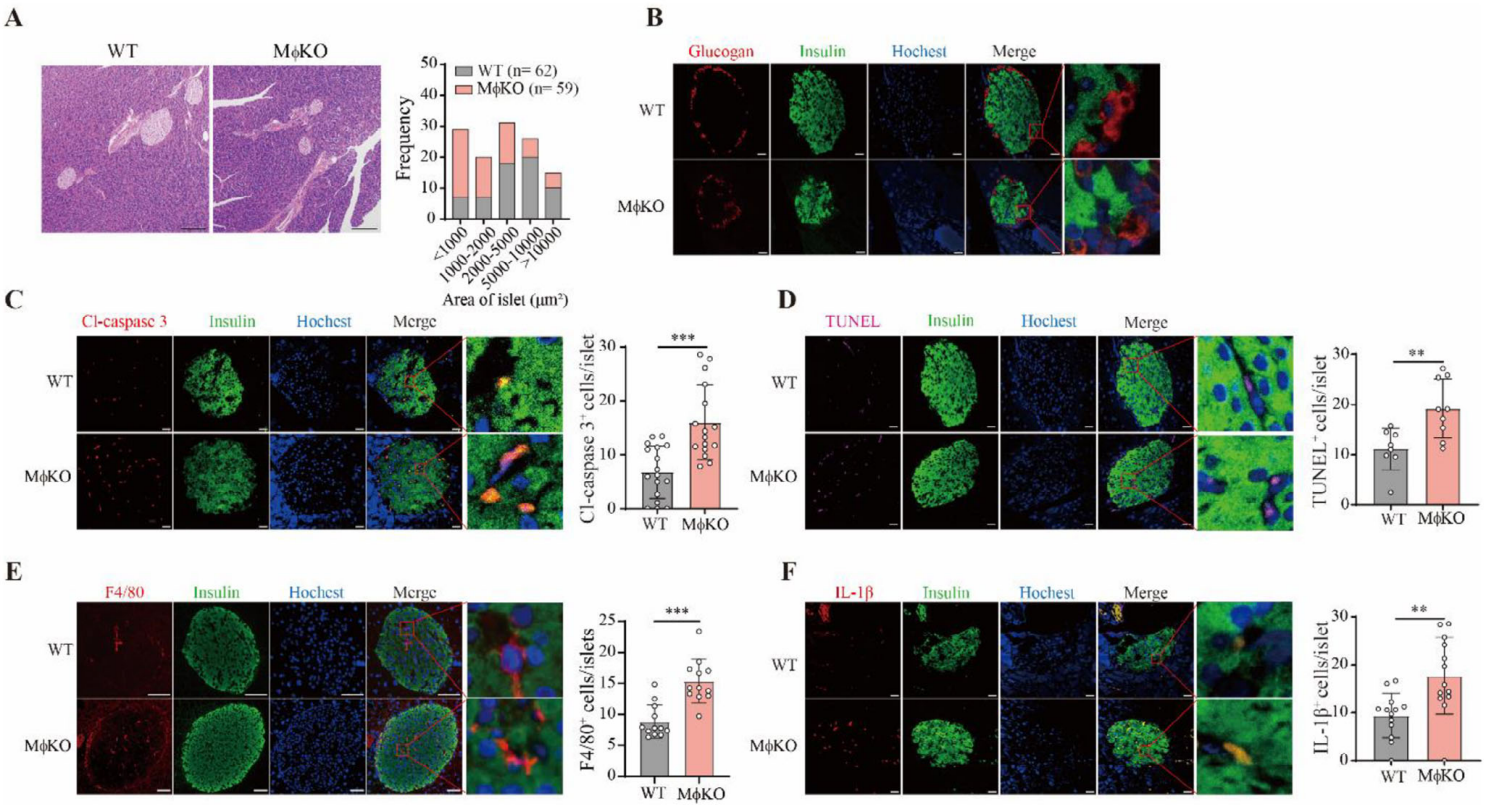
MCT1 deletion in macrophages exacerbates islet cell death and increases macrophage infiltration in HFD mice. A) Representative H&E staining of pancreas in WT and MφKO mice with HFD for 16 weeks. Statistics of the area of islets were analyzed, n = 62 for WT mice and n = 59 for MφKO mice. Six mice from each group were used in the analysis. B–F) Immunofluorescence staining of pancreatic paraffin sections prepared from WT and MφKO mice with HFD. Representative images of insulin and glucagon staining (B); insulin and cleaved‐caspase3 staining (C), insulin and TUNEL staining (D), insulin and F4/80 staining (E), and insulin and IL‐1β staining. Statistics of the ratio as compared to insulin staining are shown, cleave‐caspase3 (n = 17 for WT, n = 16 for MφKO), F4/80 (n = 12 for both groups), TUNEL (n = 7 for WT, n = 9 for MφKO), and IL‐1β (n = 13 for both groups). The enlarged images are shown in the right panels. Data are presented as mean ± SD with each point representing a biological replicate and n representing the number of biological replicates. The *p* values were calculated using unpaired, two‐sided Student's *t*‐test. **p < 0.01, ***p < 0.001. Scale bar: 100 µm (A), 20 µm (B–D, F), 50 µm (E).

As glucose intolerance and insulin secretion were compromised in the MφKO mice, we analyzed the pancreatic islets of these mice. First, we found that the area of islets was reduced in the MφKO mice through hematoxylin‐eosin staining (HE) (**Figure** **5**A). Roughly 37.2% of islets in MφKO mice had an area lower than 1 × 10^3^ µm^2^, compared to 11.3% in wild type mice (Figure 5A). However, the overall morphology and the distribution patterns of β cells and α cells of the islets appeared not different between the two groups of mice (Figure 5B). We next analyzed the level of cell death in the islets through immunofluorescent staining with cleaved‐caspase3 and TUNEL. We found that the percentage of cleaved‐caspase 3 positive cells was significantly increased in the islets of MφKO mice (16.01% in MφKO mice compared to 6.8% in wild type mice) (Figure 5C). The percentage of TUNEL‐positive cells was also elevated in MφKO mice (20.03% MφKO mice compared to 10.47% in wild type mice) (Figure 5D). Next, we analyzed macrophages in the islets through immunofluorescent staining with a macrophage marker F4/80. The number of macrophages in the islets was significantly increased by *Slc16a1* deletion in macrophages, with 10.6% in wild type mice and 20.4% in MφKO mice (Figure 5E). Consistently, the percentage of IL‐1β‐positive cells was significantly elevated in the islets of MφKO mice (Figure 5F). Collectively, these data suggest that loss of MCT1 in macrophages aggravates apoptosis and inflammation in the islets of MφKO mice under HFD condition.

### 2.6 Islet Isolation and Co‐Culture Experiments Reveal that Loss of MCT1 in Macrophages Aggravates Apoptosis of b Cells

**Figure 6:**
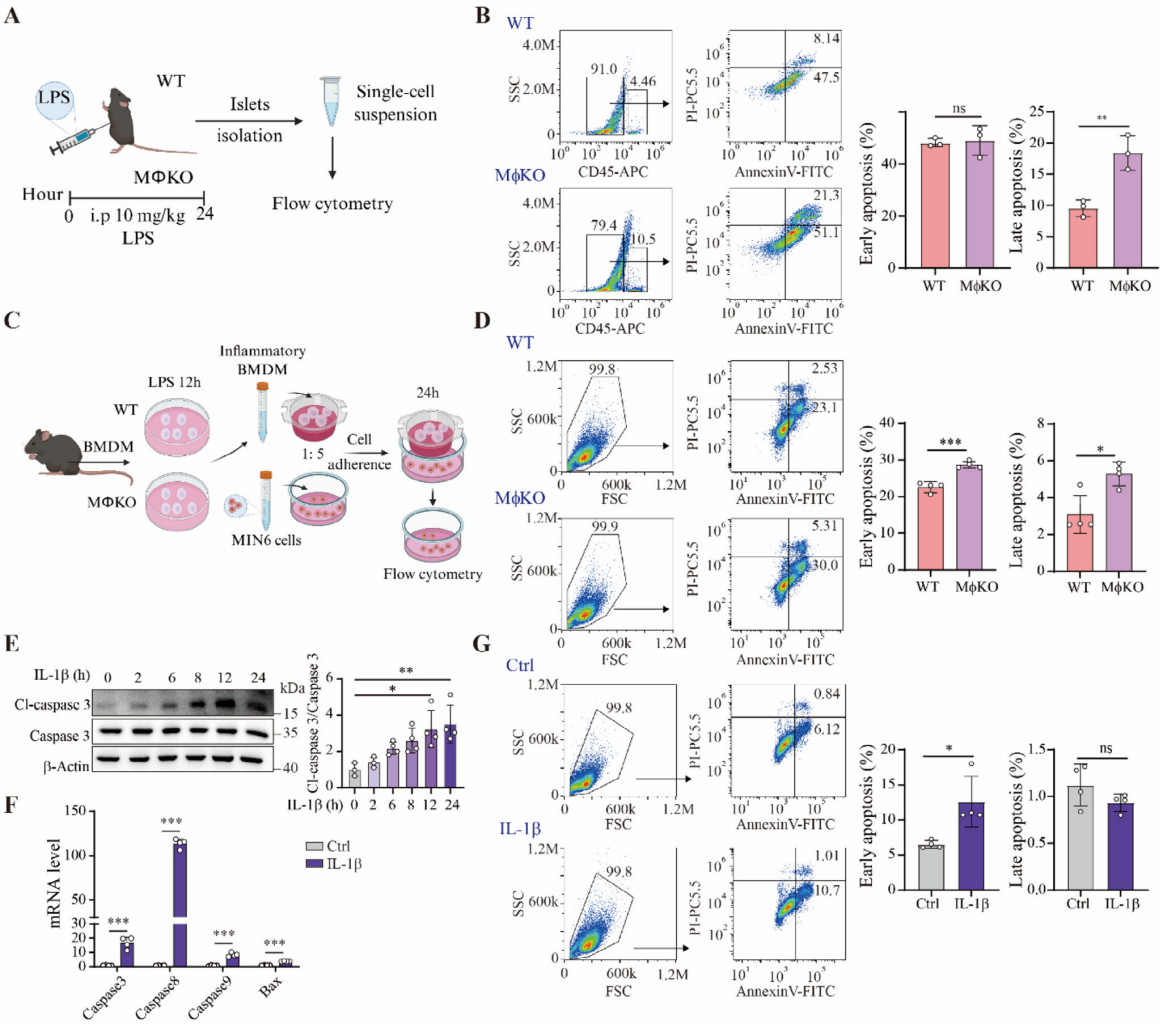
Blocking MCT1 together with LPS treatment in macrophages aggravates apoptosis of islet β cells and MIN6 cells. A) Schematic of experimental design for B. The mice underwent intraperitoneal injection with LPS 10 mg kg^−1^ for 24 h before islet isolation. B) Flow cytometry plot of the isolated islets. The percentage of Annexin V^+^PI^‐^ early apoptosis cells and Annexin V^+^PI^+^ late apoptosis cells are measured for CD45^‐^ cells, n = 3 per group. C) Schematic of experimental design for D. D) LPS‐treated BMDMs were co‐cultured with MIN6 cells in different chambers at a ratio of 1 to 5. Representative flow cytometry plot and ratio of Annexin V^+^PI^‐^ early apoptosis cells and Annexin V^+^PI^+^ late apoptosis cells are shown, n = 4 mice per group. E) Western boltting analysis of MIN6 cells treated with 10 ng mL^−1^ IL‐1β for different times. Quantitation result normalized to β‐actin is shown in the right panel, n = 4 per group. F) The mRNA levels of apoptosis‐related genes in MIN6 cells treated with IL‐1 β for 24 h, n = 4 per group. G) MIN6 cells were treated with 10 ng mL^−1^ IL‐1β for 24 h. Representative flow cytometry plot and ratio of early and late apoptosis cells are shown, n = 4 per group. Data are presented as mean ± SD with each point representing a biological replicate and n representing the number of biological replicates. Data are representative of two independent experiments (B, D) and representative of three independent experiments (E, F). The *p* values were calculated using unpaired, two‐sided Student's *t*‐test (B, D, G, F) or one‐way ANOVA with Tukey's honest significant difference (HSD) pose hoc analysis (E). *p < 0.05, **p < 0.01, ***p < 0.001, ns for non‐significant. Images in A and B were created in BioRender. s²s², –H. (2025) https://BioRender.com/vczuh56.

As deletion of *Slc16a1* in macrophages aggravates HFD‐induced glucose intolerance, reduces insulin secretion, and increased apoptosis/inflammation in the islets, we hypothesized that MCT1 in macrophages could impact apoptosis of β cells. We peritoneally injected LPS in 8‐week‐aged mice to generate an inflammatory state and isolated islets for flow cytometry analysis (**Figure** **6**A). As expected, loss of MCT1 gave rise to an increase in the percentage of CD45^+^ cells (Figure 6B). In the isolated islets, the percentage of late‐stage apoptotic islet cells was significantly elevated in MφKO mice (Figure 6B). Next, we isolated BMDMs from both the WT and MφKO mice and treated the macrophages with LPS, and then co‐cultured the macrophages with mouse β cell line MIN6 cells (Figure S10, Supporting Information). We found that co‐culture of *Slc16a1*‐deleted macrophages with MIN6 cells significantly elevated apoptosis of MIN6 cells (Figure S11, Supporting Information). We also analyzed the inflammatory cytokine levels in this co‐culture experiment. We found that the levels of secreted pro‐inflammatory cytokines tended to be elevated when MIN6 cells were co‐cultured with LPS‐activated macrophages in which MCT1 was deleted (Figure S11A, Supporting Information). In addition, the extracellular lactate level was increased by LPS treatment when MIN6 cells were co‐cultured with macrophages (Figure S11B, Supporting Information). In particular, loss of MCT1 further increased the extracellular lactate level (Figure S11B, Supporting Information). We repeated the co‐culture experiment by culturing BMDMs and MIN6 cells in separate chambers (Figure 6C). This co‐culture experiment also revealed that loss of MCT1 in macrophages promoted MIN6  cell apoptosis (Figure 6D), further indicating that the factors secreted by MCT1‐deleted macrophages may mediate apoptosis of MIN6 cells.

We discovered that IL‐1β is one of the major inflammatory cytokines secreted by pro‐inflammatory macrophages with MCT1 loss (Figure 1). Numerous previous studies also demonstrated that IL‐1β can induce β cell apoptosis.^[^ ^42^, ^43^ ^]^ We posited that the increased IL‐1β production of macrophages upon MCT1 loss might mediate β cell apoptosis. We treated MIN6 cells with IL‐1β and found that IL‐1β treatment led to a significant upregulation of cleaved‐caspase3 protein (Figure 6E and Figure S12, Supporting Information) and expression of an array of apoptosis‐related genes in MIN6 cells (Figure 6F). Similarly, the percentage of apoptotic MIN6 cells was increased by IL‐1β treatment (Figure 6G). Collectively, our results suggest that pro‐inflammatory macrophages with *Slc16a1* deletion exacerbate β cells apoptosis, at least partly impacted by IL‐1β.

### 2.7 Lactate Suppresses Insulin Secretion of β Cells

**Figure 7:**
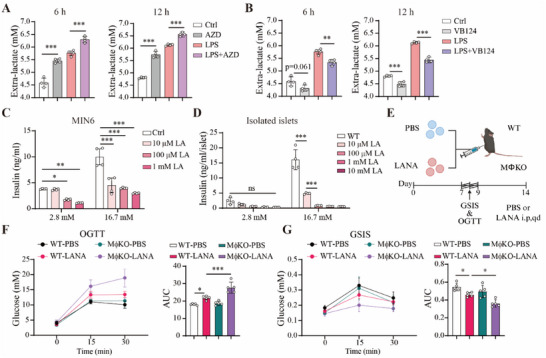
Lactate suppresses insulin secretion in MIN6 cells, isolated islets and in vivo. A) Extracellular lactate concentration in BMDMs treated with LPS in the presence or absence of AZD3965 for 6 h or 12 h, n = 4 per group. B) Extracellular lactate concentration in BMDMs were treated with LPS in the presence or absence of 10 µM VB124 for 6 h or 12 h, n = 4 per group. C) GSIS in MIN6 treated with different concentrations of lactate for 24 h, n = 4 per group. D) GSIS in isolated islets treated with sodium lactate at different concentrations, n = 4 per group. Each point represents as a biological replicate and n represents the number of biological replicates. E) Schematic of experimental design for F to G. The mice underwent daily intraperitoneal injection of sodium lactate (LANA) at 500 mg kg^−1^, pH = 7.4. The injection was stopped from day 7 to day 9, and restarted at day 10. The image was created in BioRender. s²s², –H. (2025) https://BioRender.com/vczuh56. F, G) Oral glucose tolerance test (OGTT) and GSIS of the mice at day 8, n = 5 mice per group. AUC is shown on the right. Data are presented as mean ± SD. Data are representative of three independent experiments (A–C) and representative of two independent experiments (D). The *p* values were calculated using one‐way ANOVA with Tukey's honest significant difference (HSD) pose hoc analysis (A, B, F, G) or two‐way ANOVA with Tukey's HSD pose hoc analysis (C, D). *p < 0.05, **p < 0.01, ***p < 0.001, ns for non‐significant.

M1‐like macrophages have an increased expression of MCT1 and decreased expression of MCT4 (Figure 1A,C). Based on our model, the altered expression of MCT1 and MCT4 would lead to an increase of lactate efflux to the extracellular space (Figure 3J). Consistent with this model, we found that LPS could increase extracellular lactate level of BMDMs and that such an increase was further enhanced by inhibition of MCT1 with AZD3965 (**Figure** **7**A, at both 6 and 12 h). In contrast, inhibition of MCT4 with VP124 decreased LPS‐induced elevation of the extracellular lactate level (Figure 7B). Considering that deletion of MCT1 can promote M1‐like polarization of macrophages, we postulated that the increased extracellular lactate level via MCT1 loss might also alter β cell functions. Intriguingly, we found that glucose‐stimulated insulin secretion was reduced by lactate treatment in MIN6 cells (Figure 7C). Furthermore, we isolated islets from 12‐week‐old male C57BL/6J WT mice and analyzed whether lactate treatment was able to alter insulin secretion. GSIS assay with the isolated islets demonstrated that lactate at concentration as low as 10 µM was able to inhibit insulin secretion (Figure 7D). Therefore, these results provided convincing evidence that lactate at sub‐physiological concentrations is able to suppress insulin secretion in β cells.

We also analyzed the in vivo effect of lactate treatment on glucose homeostasis and islet (Figure 7E). High concentration of sodium lactate (LANA, pH = 7.4, 500 mg kg^−1^) was injected intraperitoneally to the mice for 7 days. Administration of sodium lactate aggravated glucose tolerance, and such aggravation was worsened by MCT1 deletion (Figure 7F). Meanwhile, glucose‐stimulated insulin secretion was reduced by sodium lactate, and such reduction was worsened by MCT1 loss (Figure 7G). Therefore, these in vitro and in vivo data suggest that lactate is able to suppress insulin secretion of β cells.

### 2.8 Lactate Inhibits Insulin Secretion of β Cells through Activating G‐Protein Coupled Receptor GPR81

**Figure 8:**
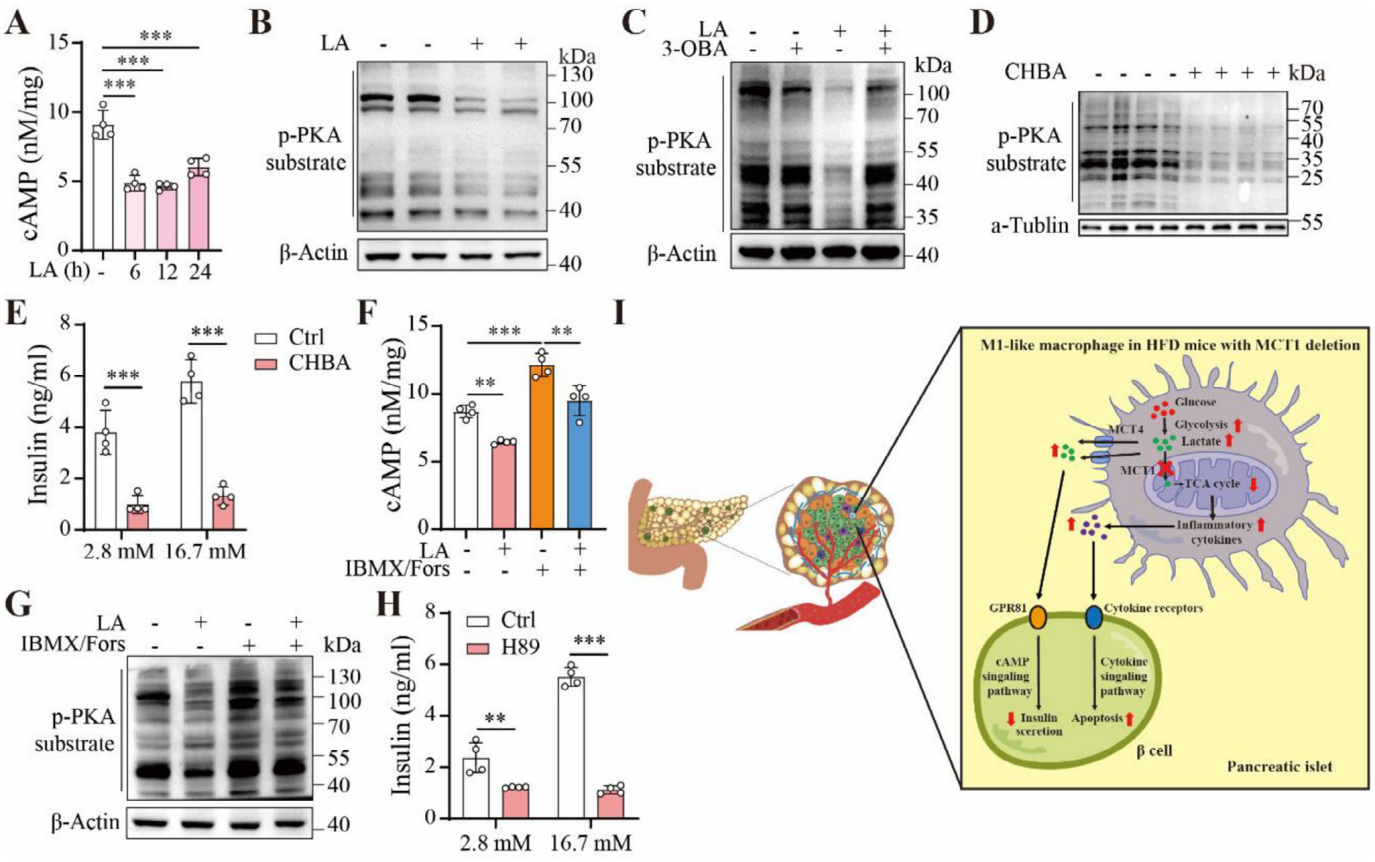
Lactate suppresses insulin secretion of β cells via GPR81‐cAMP‐PKA signaling pathway. A) Levels of intracellular cAMP in MIN6 cells treated with 100 µM lactate for different times, n = 4 per group. B) Western blotting analysis of PKA substrate phosphorylation in MIN6 cells treated with 100 µM lactate for 24 h. C) Western blotting analysis of PKA substrate phosphorylation in MIN6 cells treated with 100 µM lactate in the presence or absence of 50 mM 3‐hydroxybutyric acid (3‐OBA) for 24 h. D) Western blotting analysis of PKA substrate phosphorylation in MIN6 cells treated with 50 nM CHBA for 24 h. E) GPR81 agonist CHBA decreases insulin secretion of β cells. MIN6 cells were treated with 50 nM CHBA for 24 h, followed by GSIS, n = 4 per group. F) The level of intracellular cAMP in MIN6 cells treated with 100 µM lactate and IBMX (100 µM)/forskolin (25 µM) for 24 h, n = 4 per group. G) Western blotting analysis of MIN6 cells as in F. H) PKA inhibitor decreases insulin secretion in β cells. MIN6 cells were treated with 25 nM H89 for 24 h, followed by GSIS, n = 4 per group. I) A proposed model to depict how MCT1/MCT4 regulates macrophage polarization and impacts survival and insulin secretion of β cells in islets. Data are presented as mean ± SD with each point representing a biological replicate and n representing the number of biological replicates. Data are representative of two independent experiments (A, C–G) and representative of four independent experiments (B). The *p* values were calculated using one‐way ANOVA with Tukey's honest significant difference (HSD) pose hoc analysis (A, F) or two‐way ANOVA with Tukey's HSD pose hoc analysis (E, H). **p < 0.01, ***p < 0.001.

Next, we investigated the molecular mechanism underlying lactate‐induced suppression of insulin secretion in β cells. Bearing in mind that G‐protein coupled receptor GPR81 (also known as HCAR1, coupled to inhibition of cAMP‐PKA pathway) has been previously characterized to be a specific receptor for lactate^[^ ^18^, ^44^ ^]^ and that MCT1 is disallowed in β cells,^[^ ^45^ ^]^ we focused our studies on GPR81. Treatment of MIN6 cells with lactate decreased the intracellular cAMP level (**Figure** **8**A). Consistently, lactate treatment inhibited phosphorylation of PKA substrates (Figure 8B). To verify whether GPR81 is involved in the action of lactate, we analyzed the effect of a GPR81 inhibitor 3‐hydroxybutyric acid (3‐OBA) in β cells. As expected, lactate‐mediated reduction of PKA substrate phosphorylation in MIN6 cells was cancelled out by 3‐OBA treatment (Figure 8C). Importantly, we found that activating GPR81 by its agonist CHBA decreased PKA substrate phosphorylation (Figure 8D) and suppressed insulin secretion (Figure 8E) in MIN6 cells. We also analyzed the effect of increasing intracellular level of cAMP through treatment with IBMX and forskolin. As expected, lactate‐mediated reduction of intracellular cAMP level was nullified by IBMX/forskolin (Figure 8F). Lactate‐mediated reduction of PKA substrate phosphorylation was cancelled out by IBMX/forskolin (Figure 8G). Furthermore, inhibition of PKA by H89 decreased insulin secretion in MIN6 cells (Figure 8H). Collectively, these data indicated that lactate‐induced insulin secretion in MIN6 cells are mediated by the GRP81‐cAMP‐PKA signaling pathway. Combining these results with our earlier finding that IL‐1β induced apoptosis of MIN6 cells (Figure 6E‐G), we propose a model to summarize the effect of MCT1 deletion in macrophages on β cell functions (Figure 8I). MCT1 deletion in macrophages promotes M1‐like polarization, leading to increased secretion of pro‐inflammatory factors and elevated lactate efflux to the extracellular space. The increased release of IL‐1β and likely other pro‐inflammatory cytokines leads to MIN6 β‐cell death via their corresponding cytokine receptors. Meanwhile, the increase of lactate in the microenvironment suppresses insulin secretion of β cells via GRP81‐cAMP‐PKA signaling pathway. Such paradigm thus illustrates a unique function of macrophage MCT1 in regulating β cells and glucose homeostasis.

## 3 Discussion

In this study, we identified an important function of lactate flux through MCT1 and MCT4 in governing macrophage polarization. It was well known that metabolic reprogramming dictates macrophage polarization.^[^ ^1^, ^2^, ^3^, ^4^, ^28^ ^]^ M1‐like macrophages are featured by an increase in glycolysis and reduction of mitochondrial activity, while M2‐like macrophages are characterized by a decrease in glycolysis and an increase in mitochondria oxidation.^[^ ^7^, ^8^ ^]^ Our study reveals that distinct lactate shuttles through MCT1 and MCT4 are fundamental in modulating macrophage polarization (Figure 3J). We propose that MCT4 is mainly localized in the plasma membrane and mediates the intercellular lactate shuttle, while MCT1 is mainly localized on the mitochondrial membrane to mediate the intracellular lactate shuttle to enhance lactate flux from cytoplasm to the mitochondria. In M1‐like macrophages, a decrease in mitochondrial localization of MCT1 reduces transport of lactate to the mitochondria, contributing to an increase in cytoplasmic lactate, which is further increased by elevated glycolysis. Therefore, export of lactate by MCT4 to the extracellular space would be further enhanced in M1‐like macrophages. In contrast, M2 polarization of the macrophages have an increase in mitochondrial localization of MCT1, leading to increased lactate transport to the mitochondria and consequent enhancement of the TCA cycle. We also propose that the difference in lactate transport to the mitochondria via MCT1 contributes to the alterations of mitochondrial function during macrophage polarization, as lactate can serve as a fuel to the TCA cycle.^[^ ^12^ ^]^

In this study, our focus was on the regulation of macrophage functions by lactate transport mediated by MCT1 and MCT4. MCT1‐4 are capable of transporting lactate and pyruvate, whereas MCT7 is responsible for transporting ketone bodies.^[^ ^46^ ^]^ Additionally, MCT4 has been found to transport pyruvate at physiological concentrations.^[^ ^28^ ^]^ Consequently, we cannot entirely eliminate the possibility that pyruvate transported by MCT1 and/or MCT4 plays a role in the regulation of macrophage polarization. In our research, we propose that MCT4 primarily participates in the intercellular lactate shuttle, while MCT1 is mainly engaged in the intracellular lactate shuttle to the mitochondria. Notably, lactate transported to the mitochondria via MCT1 serves as a fuel source for the TCA cycle. Similarly, pyruvate transported to the mitochondria by MCT1, if present, also supplies energy to the TCA cycle. Thus, the ultimate outcomes of both pyruvate and lactate transport are similar, namely, to fuel the TCA cycle. In other words, the absence of MCT1 would decrease the fuel supply for the TCA cycle, regardless of whether lactate or pyruvate is transported. Blocking MCT1 would lead to a decrease in mitochondrial oxidative phosphorylation and promotes the M1‐like polarization of macrophages. Nevertheless, the question of whether MCT1 is directly involved in the transport of pyruvate to the mitochondria represents an intriguing issue that warrants further analysis in future studies.

It was found that lactate regulates macrophage metabolism via epigenetic modulation through lactylation of histone.^[^ ^14^ ^]^ Histone lactylation is induced during M1 macrophage polarization, consistent with the notion that glycolysis is elevated in these cells. In the late phase of M1 polarization, histone lactylation is implicated in the expression of homeostatic genes that are implicated in would healing.^[^ ^14^ ^]^ We propose that the lactylation of histones and likely other proteins in response to elevated lactate levels in M1 like macrophages plays a role in an adaptive cellular response by modifying protein functions and gene expression. Moreover, alterations in lactate transport into the mitochondria via MCT1 during macrophage polarization could serve as a driving force for changing mitochondrial respiration, given that lactate can act as a fuel for the TCA cycle.

One of the key discoveries in this study is that MCT1 is localized in the mitochondria of macrophages, refuting the traditional concept that MCT1 is only localized in the plasma membrane to mediate lactate transport across cell membrane. Our study corroborates the concept of an intracellular lactate shuttle, as proposed by Brooks.^[^ ^11^ ^]^ In theory, both glycolysis end‐products pyruvate and lactate can be transported into the mitochondria via different transporters, that is, mitochondria pyruvate carrier (MPC) and MCT1, respectively. However, as cellular lactate dehydrogenase highly favors conversion of pyruvate to lactate,^[^ ^11^ ^]^ lactate transported to the mitochondria by MCT1 might be a major fuel source to feed the TCA cycle. This concept is also supported by a recent report showing that MCT1 in oxidative muscle fibers is involved in intracellular lactate shuttle.^[^ ^32^ ^]^ In this study, we found that M2‐like macrophages, upon treatment with IL‐4, induces MCT1 expression and its localization to the mitochondria. We propose that the signaling pathway initiated by IL‐4 would trigger such changes of MCT1. This is an important issue that needs to be addressed in the future.

Our study also provides additional evidence that the interaction of macrophages with β cells in islets is important in maintaining islet function and glucose homeostasis. It was previously discovered that there are two types of resident macrophages in the islet with distinct anatomical distribution and functions.^[^ ^47^ ^]^ It was found that obesity induces the local expansion of intra‐islet macrophages, which impair β cell function in a cell‐cell contact‐dependent manner.^[^ ^47^ ^]^ In addition, functional interactions among macrophages, neutrophils and β cells in the islets are involved in ER stress‐induced cell death.^[^ ^48^ ^]^ Our study shows that blocking MCT1 in macrophages regulates β cell functions through two distinct mechanisms, as illustrated in Figure 8I. The first mechanism involves the enhancement of M1‐like polarization resulting from MCT1 deficiency that leads to suppression of TCA cycle and mitochondrial respiration. As a consequence, multiple pro‐inflammatory cytokines, such as IL‐1β, are released. These cytokines initiate a cascade of events that lead to β cell death. The second mechanism is associated with an increase in the local extracellular lactate concentration. In M1‐like macrophages, glycolysis is stimulated, and lactate efflux is increased due to elevated expression of MCT4. The elevated extracellular lactate can suppress insulin secretion of β cells through GPR81‐cAMP‐PKA signaling pathway. In summary, when MCT1 is absent in macrophages, the combined actions of cytokines and lactate synergistically cause dysfunction of β cells in obese mice in which chronic inflammation in islets is exacerbated by obesity (Figure 8I).

Our study reveals for the first time that GPR81 is involved in lactate‐mediated suppression of insulin secretion in β cells. It has long been known that MCT1 is disallowed in β cells.^[^ ^45^ ^]^ Surprisingly, we found β cells are highly sensitive to lactate. Lactate at 10 µM could also reduce high‐glucose induced insulin secretion in MIN6 cells and isolated mouse islets (Figure 7C,D). The blood concentration of lactate is ≈1 mM under resting conditions. We speculate that the lactate level in the microenvironment of islets should be kept low, although the underlying mechanism still awaits investigation. Nevertheless, we found that GPR81 is involved in lactate‐induced dysfunction of β cells. GRP81 is a G_i_‐couple GPCR, and its function as a lactate receptor was originally discovered in adipocytes, where it enhances the inhibitory effect of insulin on lipolysis.^[^ ^18^, ^44^ ^]^ Consistently, we also found that lactate inhibits cAMP production and PKA activation in β cells. At present we cannot rule out the possibility that lactate may regulate other functions of β cells through GPR81. Based on our findings, antagonizing the GRP81 signaling pathway of β cells might be an intriguing strategy for preserving the normal functions of β cells for the treatment of diabetes in the future. This idea is in line with recent discoveries that show inhibiting GPR81 alleviates liver fibrosis and attenuates tumor cachexia.^[^ ^49^, ^50^ ^]^

Intriguingly, it is noteworthy that our findings about the suppressive activity of lactate on insulin secretion via GRP81 may provide a mechanistic explanation to earlier observations that acute exercise significantly reduces insulin secretion.^[^ ^51^ ^]^ Acute exercise rapidly increases the blood lactate level. A lot of earlier physiology studies have found that exercise rapidly reduces insulin secretion, a question that has puzzled the field for a long time. A few studies have indicated that activation of α2‐adrenergic receptor in β cells is partially involved in exercise‐induced suppression of insulin secretion.^[^ ^52^ ^]^ Interestingly, α2‐adrenergic receptor is coupled to G_i_, resulting in reduction of intracellular cAMP. This observation is consistent with our results showing that lactate can activate GPR81 in MIN6 β cells to decrease intracellular cAMP level. In other word, our results indicate that exercise‐induced suppression of insulin secretion is partly mediated by lactate activation of GPR81.

It is notable that MCT1 can participate in either intercellular lactate shuttle or intracellular lactate shuttle dependent on cellular context. In macrophages, as shown in this study, MCT1 is mainly responsible for lactate shuttle to the mitochondria. In adipocytes, MCT1 is mainly involved in lactate efflux.^[^ ^29^ ^]^ Under obese conditions, hypoxia elevates the intracellular lactate level and such elevation subsequently triggers apoptosis in adipocytes and release of pro‐inflammatory cytokines. In adipocytes lacking MCT1, lactate efflux is inhibited, exacerbating apoptosis and the secretion of pro‐inflammatory cytokines. These effects contribute to systemic insulin resistance in obese mice.^[^ ^29^ ^]^

In conclusion, our study not only underscores the crucial role of the MCT1‐mediated intracellular lactate shuttle in determining macrophage polarization but also identifies the participation of macrophage MCT1 in the functional interplay between macrophages and β cells within the islets. Additionally, our research reveals a functional significance of GPR81 in mediating lactate‐induced inhibition of insulin secretion and apoptosis of MIN6 β‐cells. These findings would enhance our comprehension of the intricate roles of lactate transport in both physiological and pathophysiological processes. As a result, they lay the groundwork for the discovery of novel strategies to manage dysfunctions in glucose homeostasis, such as those observed in diabetes.

## 4 Experimental Section

### Reagents, Kits and PCR Primers

The detailed information of reagents, kits and PCR primer sequence used in this study is given in Table S1, Supporting Information.

### Mouse Studies

Mouse were kept under specific pathogen‐free condition and they were all on C57BL/6J background. The Slc16a1^f/f^ mice were developed by Shanghai Model Organisms Center (Shanghai, China), and Lyz2^Cre^ mice were purchased from the same institution. Slc16a1^f/f^ mice (called WT) were crossed with Lyz2^Cre/‐^ mice to obtain Slc16a1^f/f^ Lyz2^Cre/‐^ mice (called MφKO). Age‐matched littermates of both genders with wild‐type Slc16a1^f/f^ genotype were used as controls. The mouse experiments and normal feeding were conducted using standard chow, except for those involving a high‐fat diet (HFD). All animal experimental protocols were approved by Institutional Animal Care and Use Committee Institutional Animal Care and Use Committee of Shanghai Institute of Nutrition and Health, Chinese Academy of Sciences (CAS) with an approval number SINH‐2024‐CY‐1.

### Primary Cell Culture and Islet Isolation

For mouse bone‐marrow derived macrophage (BMDM) preparation, bone marrow cells were flushed from femur and tibia bones of 6–8 weeks old mice and were cultured in RPMI Medium 1640 (Gibco) supplemented with 10% fetal bovine serum (FBS), 100 U mL^−1^ penicillin and streptomycin, and 30% L929 supernatant (BMDM growth medium) for 5–6 days as indicated for individual experiments. Islets were isolated from the mice and the experimental methods are described in the method details section. Islets were cultured in DMEM with 15% FBS, 100 U mL^−1^ penicillin and streptomycin.

### Cell Lines and Cell Culture

The cell lines used in this study included human embryonic kidney cell line HEK293T, mouse macrophage cell line RAW264.7 (provided by Dr. Wei Lv, Shanghai Institute of Nutrition and Health, Chinese Academy of Science), mouse fibroblast cell line L929 (provided by Dr. Ming Lu, Shanghai Institute of Nutrition and Health, Chinese Academy of Science), mouse islet beta cell line MIN6. HEK293T and L929 cells were cultured in DMEM with 100 U mL^−1^ penicillin/streptomycin and 10% FBS; RAW264.7 cells were cultured in RPMI 1640 with 100 U mL^−1^ penicillin/streptomycin and 10% FBS; MIN6 cells were culture in DMEM with 100 U mL^−1^ penicillin/streptomycin and 15% FBS. All cell lines were cultured at 37 °C with 5% CO_2_.

### LPS‐Induced Acute Inflammation of Mice

8‐week‐old female mice received intraperitoneal injection of 10 mg kg^−1^ LPS (Served on 1× PBS, sterilized by filtration, 1× PBS as vehicle) for 24 h.

### Mouse Experiment with High Fat Diet

7 week‐aged male mice were changed to high‐fat diet (HFD) with 60% of kilocalories from fat (cat. no. D12492; Research Diets) for 16 weeks. At different times after HFD, following tests were performed. For measurement of fasting blood glucose (at 1 month after HFD), the mice were fasted for 16 h. Blood glucose concentration was measured by bleeding from the tail vein using a handheld glucose meter (Contour Next, Bayer). For glucose (GTT, at 12th week after HFD) and insulin tolerance (ITT, at 13th week after HFD) tests, mice were fasted for 16 h (for GTT) or 8 h (for ITT) prior to intraperitoneal administration of 2 g kg^−1^ glucose (Sigma) or 1 unit kg^−1^ insulin, respectively. Blood glucose concentration was measured at 0, 15, 30, 60, 90, and 120 min after injection for both GTT and ITT. For glucose‐stimulated insulin secretion (GSIS), the HFD mice at 14th week were fasted for 16 h before intragastric administration of glucose at a dose of 3 g kg^−1^. Blood samples were collected at 0, 15 and 30 min for subsequent determination of serum insulin levels. The body weight and food intake were recorded on a weekly basis prior to the alteration of the high‐fat diet. The fat mass and lean mass of mice were measured at the endpoint time, analyzed by Nuclear Magnetic Resonance (NMR).

### Lactate‐Induced Apoptosis in Mouse Islets

For analysis of the effect of lactate on islet apoptosis, 8–9 week‐aged male mice were intraperitoneally injected with 500 mg kg^−1^ of sodium lactate (at pH 7.4) daily for a period of 2 weeks. The injection of sodium lactate was discontinued on days 7–9 for the GSIS and OGTT tests. Following a 16‐h fast, glucose (3 g kg^−1^) was intragastric administration. Blood samples were collected at 0, 15 and 30 min, simultaneously measuring the blood glucose concentration for subsequent determination of serum insulin levels.

### Islet Isolation

8–12 week‐aged mice were euthanized, and freshly prepared collagenase P (Roche, Indianapolis, IN) solution (0.5 mg mL^−1^) was injected into the pancreas via the common bile duct. The perfused pancreas was digested at 37 °C for 15 min, and the islets were handpicked under a stereoscopic microscope, dispersed into single cell suspensions for flow cytometry, or were cultured in complete DMEM medium.

### Live‐Cell Fluorescence Imaging

RAW264.7 cells were plated on a 35 mm 4‐chamber glass‐bottom dish. Briefly, 0.5 µg PLVX‐Fila‐Mito plasmid or PLVX‐Fila‐Cyto plasmid (from FR Biotechnology, Shanghai, China) was mixed with 50 µL jetPRIME buffer and then 1 µL of jetPRIME reagent was added according to manufacturer's protocols. The transfection mix was added to the cells in serum containing medium. After incubation for 48 h, cells were treated with LPS or IL‐4 in the presence or absence of 100 nM AZD3965, or 10 mM lactate at times as indicated in the Figure 3.

### Flow Cytometry

Freshly collected mouse blood was placed on ice and treated with RBC lysis buffer (BD Pharm Lyse) for 5 min on ice. The lysis reaction was stopped by adding 5 volumes of pre‐cooled 1 × PBS, followed by centrifugation to collect the RBC‐depleted cell pellet. The pellet was resuspended in 1 × PBS containing Fixable Viability 510 dye (1:5000 dilution) and incubated on ice for 10 min to stain dead cells. After quenching with 1 × PBS and centrifugation, the supernatant was discarded. The cells were then resuspended in 1 × PBS containing anti‐CD45‐APC‐Cy7, anti‐CD11b‐FITC, and anti‐F4/80‐PE610 antibodies (all at 1:100 dilution) and incubated on ice for 30 min. The staining was stopped by adding 1× PBS, and cells were washed three times with 1 × PBS, followed by resuspension in 100 µL 1 × PBS for flow cytometry. During analysis, debris was excluded using FSC/SSC gating. For gating, fixable Viability 510 (V510)‐negative cells were selected to remove dead cells, and CD45⁺CD11b⁺ double‐positive cells were identified as myeloid cells. Within this population, CD11b⁺F4/80⁺ cells were gated as macrophages. For analysis of islet cells, the mouse islets were digested into a single‐cell suspension. Cells were first incubated with anti‐CD45 antibody (1:100 dilution in 1 × PBS) on ice for 15 min, followed by three washes with PBS to remove unbound antibodies. The cells were then stained with Annexin V‐FITC and Propidium Iodide (PI) prepared in Annexin V binding buffer for 5 min at room temperature in the dark. The staining was terminated by adding binding buffer, and samples were immediately analyzed by flow cytometry. Cellular components were gated using FSC/SSC parameters to exclude debris. Then the CD45‐negative cells (excluding immune cells) to quadrant gating based on Annexin V‐FITC and PI signals was performed: double‐negative (Annexin V⁻/PI⁻) cells were defined as normal viable cells, Annexin V⁺/PI⁻ as early apoptotic cells, double‐positive (Annexin V⁺/PI⁺) as late apoptotic cells, and Annexin V⁻/PI⁺ as mechanically damaged cells. The same protocol was applied to MIN6 cells directly co‐cultured with BMDMs. For MIN6 cells in transwell experiments or treated with IL‐1β, the apoptosis analysis followed identical steps but omitted the CD45 staining step. Flow cytometric analysis was performed by using a Gallios flow cytometer (Beckman Coulter). Data were analyzed with FlowJo software.

### Direct Co‐Culture of MIN6 Cells with BMDMs

BMDMs isolated from 8–10 weeks WT and MφKO mice were treated with 100 ng mL^−1^ LPS for 12 h. Following the LPS treatment, BMDMs were digested and counted in order to seed the new 12 well plate with MIN6 cells at a ratio of 1:5 for co‐culturing. After incubation for 24 h, the cells were digested to single‐cell suspensions for flow cytometry analysis

### Indirect Co‐Culture MIN6 Cells with BMDMs

BMDMs isolated from 8–10 weeks WT and MφKO mice were treated with 100 ng mL^−1^ LPS for 12 h. BMDMs were then digested and counted before being seeded in the upper chamber of transwell. Simultaneously, MIN6 cells were plated on the lower chamber of transwell. Once both cell types adhered after ≈4–6 h, they were co‐cultured for an additional 24 h. MIN6 cells were then used for flow cytometry.

### shRNA Knockdown of Slc16a1 Gene

To generate the stable cell lines, the PLKO.1 lentiviral plasmids encoding Slc16a1‐specific shRNA sequence was constructed. Lentivirus was produced by co‐transfecting two lentiviral packaging vectors (pMD2.G and psPAX2) in HEK293T cells. Lentivirus supernatants were collected 48 and 72 h after transfection. RAW264.7 cells were seeded in 6 cm dishes, and were infected the supernatants (filter sterilization) and RIPM 1640 at the ratio of 1:1, with infection performed for three to four times.

### Phagocytosis Experiment

BMDMs were cultured in RPMI 1640 medium supplemented with 10% FBS, 100 U mL^−1^ penicillin, and 100 µg mL^−1^ streptomycin at 37 °C under 5% CO₂. Cells were seeded in 96‐well plates at a density of 2 × 10⁴ cells per well. After 4 h of culture, the medium was replaced with fresh RPMI 1640 medium and the cells were treated with LPS, IL‐4, AZD3965, and VB124 as indicated for 12 h. Next, the medium was removed and cells were incubated for 1 h with 100 µL PBS containing 0.08% neutral red. Cells were washed three times with PBS. Subsequently, 100 µL cell lysis buffer (acetic acid: ethanol = 1:1, v/v) was added per well and the cells were incubated at room temperature for 4 h, followed by measurement of absorbance at 540 nm using a microplate reader.

### Macrophage Migration Assay

4 × 10⁵ macrophages were seeded in the upper chamber of transwell insert. After 6 h of treatment with LPS and/or IL‐4 in the presence or absence of AZD3965 and VB124, cells were washed three times with PBS. The medium was replaced with serum‐free RPMI 1640 medium (containing the same treatment agents), and the inserts were transferred to a lower chamber plate containing RPMI 1640 medium supplemented with 10% FBS. After an additional 6‐h incubation, the cells were fixed and stained with 0.1% crystal violet (500 µL well^−1^) for 20 min at room temperature. Non‐migrated cells on the upper membrane surface were gently removed using cotton swabs. Following three PBS washes, the inserts were air‐dried. Migrated cells were quantified by counting at least three random fields per insert, with three independent replicates performed.

### GSIS for MIN6 Cells

MIN6 cell were planted in 24‐well plated and treated with lactate, 3‐OBA or not for 24 h, followed by wash with Krebs Ringer Bicarbonate buffer (KRB buffer: 2.6 mM CaCl_2_/2H_2_O, 1.2 mM MgSO_4_/7H_2_O, 1.2 mM KH_2_PO_4_, 4.9 mM KCl, 98.5 mM NaCl, and 25.9 mM NaHCO_3_, supplemented with 20 mM HEPES) for two to three times and the cells were then fasted for 0.5 h by adding the buffer solution before being further incubated for an additional hour with a glucose‐containing KRB buffer with 2.8 or 16.7 mM glucose. The insulin concentrations in the supernatant were determined using Ultrasensitive mouse insulin ELISA kits (Ezassasy).

### Lactate Detection and ELISA Assays

For measurement of intracellular lactate, the cells were digested, counted, and then suspended in 300 µL of diluent water per 10^6^ cells. This suspension underwent three cycles of freezing and thawing. Secreted lactate was directly detected using culture medium or serum samples. Lactate levels were measured using a lactate measurement kit purchased from Nanjing Jiancheng Bioengineering Institute (Nanjing, China). Intracellular ATP and cAMP were isolated using Lysing buffer (20 mM Tris HCl, PH = 7.4, 150 mM NaCl, 1 mM EDTA, 1 mM EGTA, 1% Triton X‐100) with proteinase inhibitor. The concentrations of IL‐1β, TNFα, IL‐6, IL‐10, TGF‐β1, cAMP, and ATP were determined using ELISA kits and normalized to the protein level (for cAMP and ATP).

### Mitochondria Isolation

Mitochondria purification was carried out using Mitochondria Isolation Kit protocols (MedChemExpress, HY‐K1060‐100T). Briefly, 10 cm dishes of BMDMs were washed and harvested in ice‐cold 1× PBS. Resuspended the cell in 1 ml mitochondria isolation buffer (with 1× PMSF) incubated on ice for 15 min, followed by douncing with a glass homogenizer over 25 strokes. Cell homogenate was centrifuged at 10 000 g, 4 °C for 10 min. The supernatant was transferred to another centrifuge tube and centrifuged at 5000 g at 4 °C for 10 min, the supernatant was removed to obtain mitochondria. The supernatant was removed to a new centrifuge tube and centrifuged at 12000 g at 4 °C for 10 min, the supernatant was cytoplasm fraction. As for proteinase K assay, isolated mitochondria were stored in the storage buffer. The mitochondrial suspension was divided into three aliquots: one aliquot was treated with 50 µg mL^−1^ proteinase K, another aliquot was treated with 50 µg mL^−1^ proteinase K and 1% Triton X‐100, and were incubated in a 37 °C water bath for 30 min. Subsequently, 2 mM PMSF was added to the samples, followed by incubation on ice for 5 min to inhibit protease activity.

### Seahorse Real‐Time Cell Metabolic Analysis

Oxygen consumption rate (OCR) was performed with a Seahorse XF24/XF96 Extracellular Flux Analyzer (Agilent) in BMDMs as a measure of OXPHOS. In brief, BMDMs were seeded in quadruplicate stimulated with 100 ng mL^−1^ LPS (10^5^ cells per well, XF24) or 50 ng mL^−1^ IL‐4 (10^4^ cells per well, XF96) in the presence or absence 100 nM AZD3965 for 12 h. Prior to starting the assay, cells were washed and incubated in Seahorse Assay Medium supplemented with 10 mM glucose, 1 mM sodium pyruvate and 2 mM glutamine in incubator 37 °C without CO_2_ for 45 min. Oligomycin (1uM), FCCP (6uM), rotenone(1uM) and antimycin (1uM) were injected where indicated.

### U‐^13^C_6_ Labeled Glucose Flux in BMDMs

BMDMs were stimulated with 100 ng mL^−1^ LPS for 6 h. Following this, cells were incubated for an additional 6 h in fresh glucose culture medium 4.5 g L^−1^ U‐¹^3^C₆‐glucose with AZD3965 (500 ng mL^−1^), 5 µM UK5099, or both. For sample preparation, the supernatant was discarded, and cell samples were immediately washed with ice‐cold 1 × PBS, flash‐frozen in liquid nitrogen, and submitted to Shanghai Hua Ce Medical Laboratory for metabolomic analysis. Briefly, 400 µL of 80% methanol mixed solution was added to the sample, followed by ultrasonic treatment. The sample was placed in a −80 °C freezer for half an hour, thawed at 4 °C, and centrifuged at 14 000 rpm and 4 °C for 10 min. The sample was reconstituted with 80 µL of 50% ACN (containing internal standards). Metabolic flux analysis by UHPLC‐HRMS was performed using a Thermo Fisher Scientific Ultimate 3000 Liquid Chromatography System equipped with a Waters Amide column (100 mm × 2.1 mm, 1.7 µm). The metabolites were ionized and mass spectrometry data were collected using a Thermo Fisher Scientific Orbitrap Fusion Tribrid mass spectrometer.

### Immunohistochemistry and Immunofluorescence Staining

The mouse liver samples were sectioned and fixed in 4% paraformaldehyde (PFA) for 24 h prior to paraffin embedding. Immunohistochemistry targeting F4/80 (CST) were performed by Servicebio (Wuhan, China). The pancreas was isolated from mice and fixed overnight with 4% paraformaldehyde (PFA) for paraffin embedding. The sections were dewaxed using xylene and subjected to gradient alcohol soaking. Antigen retrieval was then performed by boiling the sections in a sodium citrate buffer (pH 6.0) for 30 min. Subsequently, the sections were blocked with a solution containing 5% new goat serum and 0.1% Triton‐X100 in PBS at room temperature for 1 h. Following this, the sections were incubated overnight at 4 °C with primary antibodies, followed by incubation with corresponding secondary antibodies at room temperature for 1 h. Nuclei staining was achieved by treating the sections with Hoechst 33342 (Molecular Probes) in PBS for a duration of 8 min. Fluorescence images were captured using ZEISS (LSM880). The dilution ratios of primary antibodies and secondary antibodies used were respectively set as 1:200 and 1:500.

### Western Blotting

Total proteins were extracted from cells with RIPA lysing buffer containing 10% proteinase inhibitor cocktail and phosphatase inhibitors (Sigma‐Aldrich), and supernatant was collected after centrifugation at 13 200 rcf (g) for 10 min at 4 °C. Total lysate protein levels were quantified using a BCA Protein Assay kit (Beyotime) according to the manufacturer's protocols. Proteins were fractionated using 10% SDS‐PAGE gels and transferred to PVDF (Fisher Scientific). Membranes were probed with primary antibody at 4 °C overnight. After incubation with secondary antibody conjugated to HRP, the membranes were scanned using Tanon‐5200. The dilution ratio of the primary antibodies and secondary antibodies were 1:1000 and 1:5000, respectively.

### Quantitative Reverse Transcriptase‐Polymerase Chain Reaction Analysis

Total RNA of macrophages and MIN6 cells was extracted using TRIZOL reagent. cDNA was synthesized through reverse transcription using the FastQuant RT Kit (Tiangen, Shanghai, China). Real‐time quantitative PCR was conducted with specific primers (Table S1, Supporting Information) using the SYBR Green PCR system (TOYOBO, Tokyo, Japan). The PCR reactions were performed on an ABI QuantStudio6 system. The mRNA levels of target gene expression were normalized against the average value of β‐actin.

### Statistical Analysis

The data analysis was conducted using GraphPad Prism 9 (San Diego, CA, USA). All data were presented as mean ± SD. Unpaired, two‐sided Student's *t*‐test was used to compare 2 conditions, one‐way ANOVA with Tukey's honest significant difference (HSD) pose hoc analysis was used for comparing 3 or more conditions, two‐way ANOVA with Tukey's HSD pose hoc analysis was used for comparing 3 or more conditions with two factors. The images obtained from IHC and immunofluorescence experiments were analyzed using ImageJ software. *p* values equal to or less than 0.05 were considered statistically significant.

## Conflict of Interest

The authors declare no conflict of interest.

## Author Contributions

Y.C. and L.C. designed the experiments. L.C. performed experiments and analyzed data. Y.L., X.Z., S.Z., Z.L., C.G., X.Y., J.C., and S.W. provided technical assistance in the animal experiments. L.C. and Y.C. wrote the manuscript and prepared the figures. All authors read and approved the manuscript.

## Supporting information



Supporting Information

Supplemental Table 1

## Data Availability

The data that support the findings of this study are available from the corresponding author upon reasonable request.
