# Supplementary material for: MCT1‐mediated Lactate Shuttle to Mitochondria Governs Macrophage Polarization and Modulates Glucose Homeostasis by Affecting β Cells
Source: Adv Sci (Weinh). 2025 Jul 14;12(38):e14760. doi: 10.1002/advs.202414760 (PMC12520533; doi:10.1002/advs.202414760)
Supplement: Supplementary file 1 — Supporting Information [file ADVS-12-e14760-s002.pdf]

ADVANCED SCIENCE
Open Access

Supporting Information

for *Adv. Sci.*, DOI 10.1002/advs.202414760

MCT1-mediated Lactate Shuttle to Mitochondria Governs Macrophage Polarization and Modulates Glucose Homeostasis by Affecting β Cells

*Lingling Chen, Yijun Lin, Xinyu Zhu, Shixuan Zhuo, Zixuan Li, Cheng Guo, Xiaoyi Ye, Jinzhu Chen, Shuying Wang and Yan Chen**

MCT1-mediated lactate shuttle to mitochondria governs macrophage polarization and modulates glucose homeostasis by affecting β cells

Lingling Chen, Yijun Lin, Xinyu Zhu, Shixuan Zhuo, Zixuan Li, Cheng Guo, Xiaoyi Ye, Jinzhu Chen, Shuying Wang, and Yan Chen

Supplemental Figures

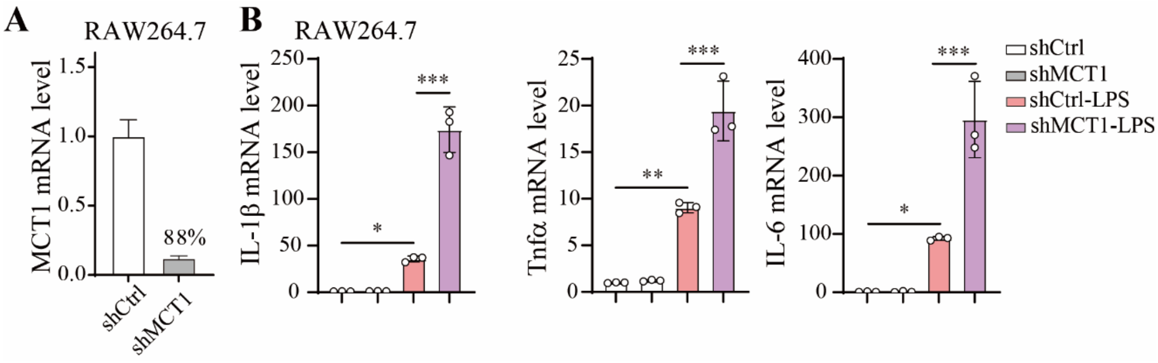

Figure S1. The efficiency of knocking down or knockout MCT1 in RAW264.7 cells

(A) Efficiency of *Slc16a1* knock down by shRNA lentivirus transfection in murine macrophage cell line RAW264.7, shown as the mRNA level of Slc16a1, n = 3.

(B) The mRNA levels of pro-inflammatory genes in *Slc16a1*-knockdown in RAW264.7 cells upon treatment with LPS (100ng/ml) for 12 h, n = 3.

Data are presented as mean ± SD with each point representing a biological replicate and n representing the number of biological replicates. Data are representative of three independent experiments. The p values were calculated using one-way ANOVA with Tukey's honest significant difference (HSD) pose hoc analysis (B). * $p < 0.05$, ** $p < 0.01$, *** $p < 0.001$. AZD, AZD3965.

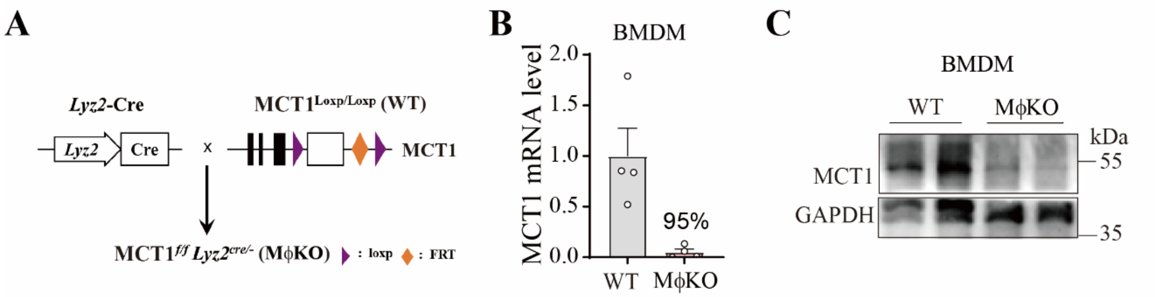

Figure S2. Generation of a mouse model with macrophage deletion of MCT1

(A) A diagram to depict the generation of macrophage-specific *Slc16a1*-deleted mice (MϕKO).

(B and C) *Slc16a1* mRNA level and MCT1 protein level in BMDM isolated from WT and MϕKO mice. The mRNA level of *Slc16a1* was reduced by 95% in the MϕKO mice.

Data are presented as mean ± SD. Data are representative of two independent experiments (B and C).

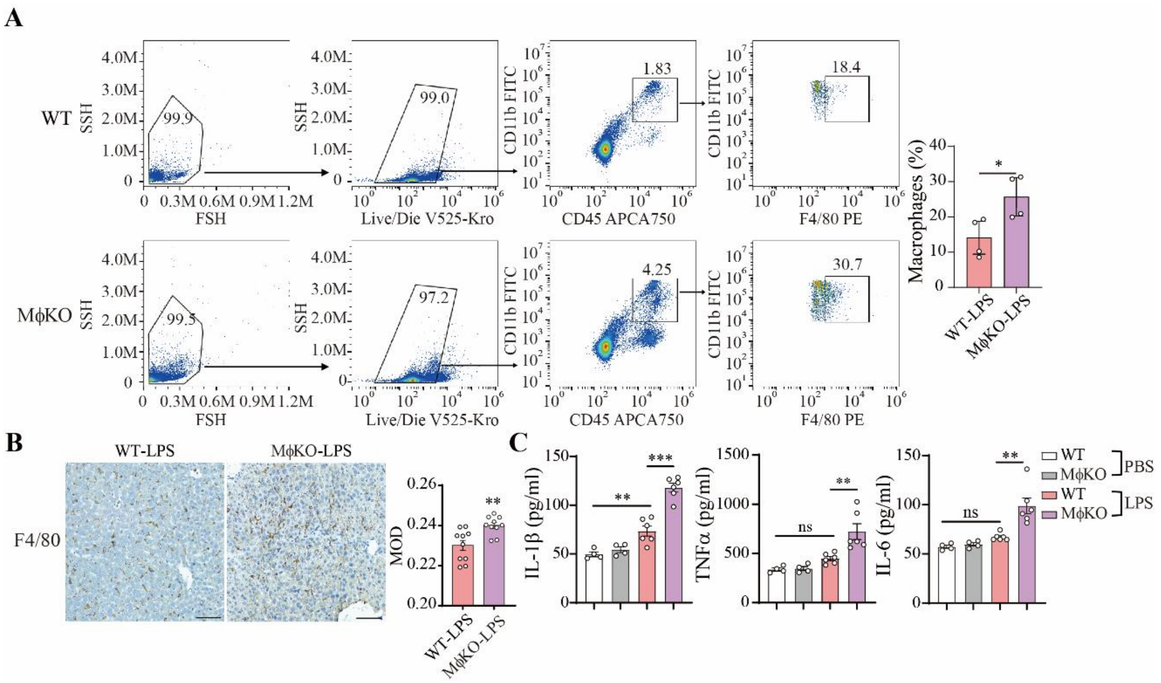

Figure S3. MCT1 deficiency in macrophages aggravated LPS-induced acute inflammation in mice

(A) Representative flow cytometry plots and ratio of $CD11c^{+}F4/80^{+}$ macrophages in blood from WT and MϕKO mice which underwent intraperitoneal injection with 10 mg/kg LPS (this concentration is used in the treatment of LPS on mice covered in this article) for 24 h, n = 4 mice per group.

(B) IHC staining of F4/80 in liver of the mice as in H. MOD = OD*staining area/total tissue area, n = 10 biological replicates. The quantitative data are shown in the right. Scale bar: 50 μm.

(C) IL-1β, TNFα and IL-6 concentration in the mouse serum (n = 4 mice for PBS-treatment and n = 6 mice for LPS-treatment).

Data are presented as mean ± SD. Data are representative of two independent experiments. The *p* values were calculated using unpaired, two-sided Student's t test (A and B) or one-way ANOVA with Tukey's honest significant difference (HSD) pose hoc analysis (C). * $p < 0.05$, ** $p < 0.01$, *** $p < 0.001$, ns for non-significant.

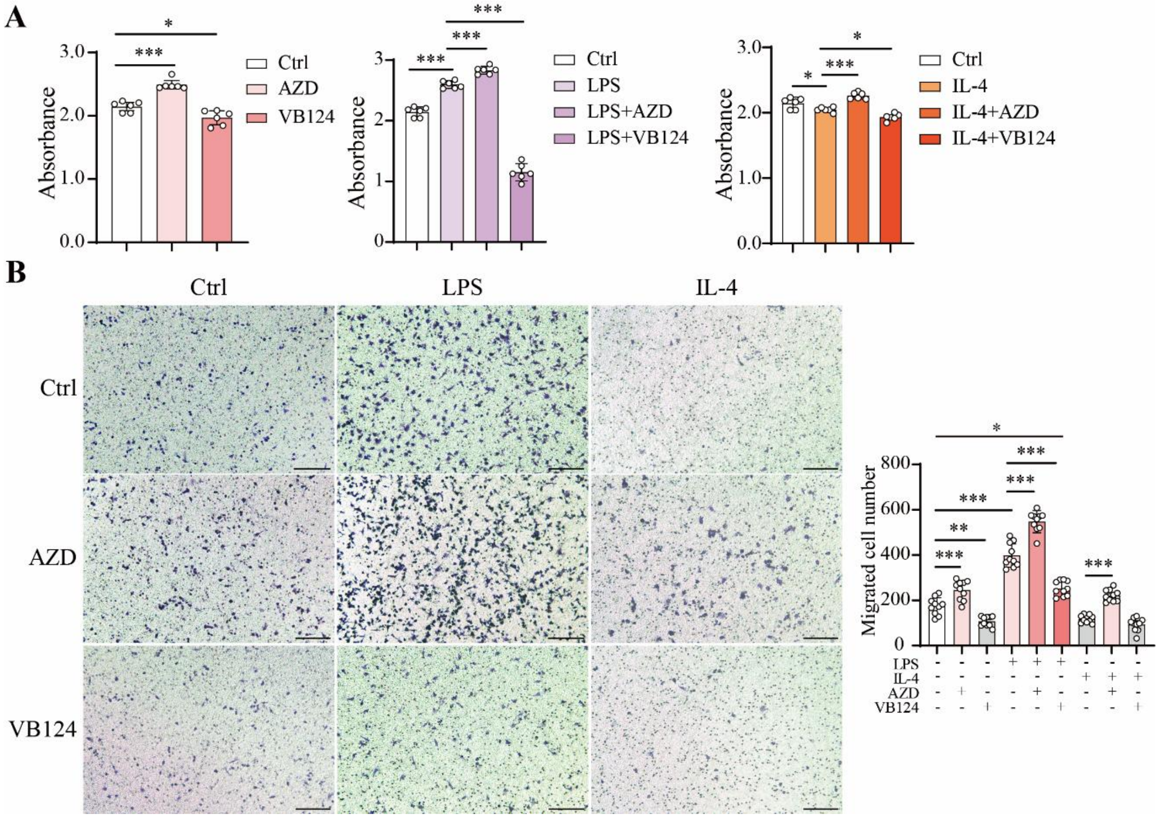

Figure S4. The effects of blocking MCT1 and MCT4 on phagocytosis and migration of macrophages

(A) Phagocytosis of macrophages as measured by absorbance of macrophages after phagocytosis of neutral red. BMDMs were treated with LPS or IL-4 in the presence or absence of AZD3965 and VB124 for 12h, followed by addition of 0.08% neutral red for 4h and then analyzed by absorbance at 540 nM, n = 6.

(B) Macrophage migration as measured by transwell assay. BMDMs were treated with LPS and IL-4 in the presence or absence of AZD3965 and VB124, respectively, n = 10. Each point represents as a biological replicate and n value represents the number of biological replicates. Scale bar: 50 μm.

Data are presented as mean ± SD. Data are representative of two independent experiments. The *p* values were calculated using one-way ANOVA with Tukey's honest significant difference (HSD) pose hoc analysis. * $p < 0.05$, ** $p < 0.01$, *** $p < 0.001$.

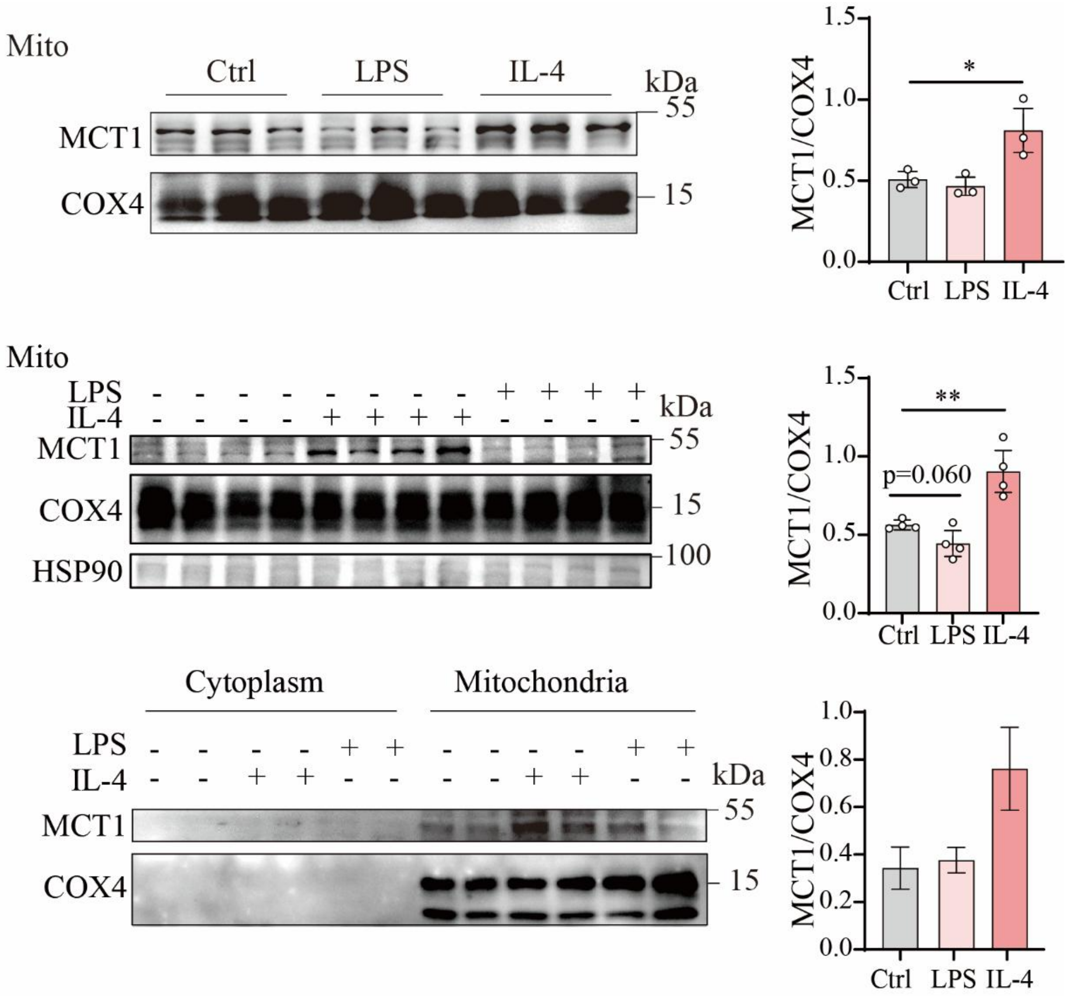

Figure S5. Results of multiple experiments to analyze how LPS and IL-4 alter MCT1 protein in mitochondrial fraction of BMDMs

Three independent experiments to analyze mitochondrial localization of MCT1 protein in BMDM were treated with LPS (100ng/ml) or IL-4 (50 ng/ml) for 12 h. Quantitation result normalized to COX4 is shown in the right panel. Each point represents as a biological repeat.

Data are presented as mean ± SD. The *p* values were calculated using unpaired, two-sided Student's t test. * $p < 0.05$, ** $p < 0.01$.

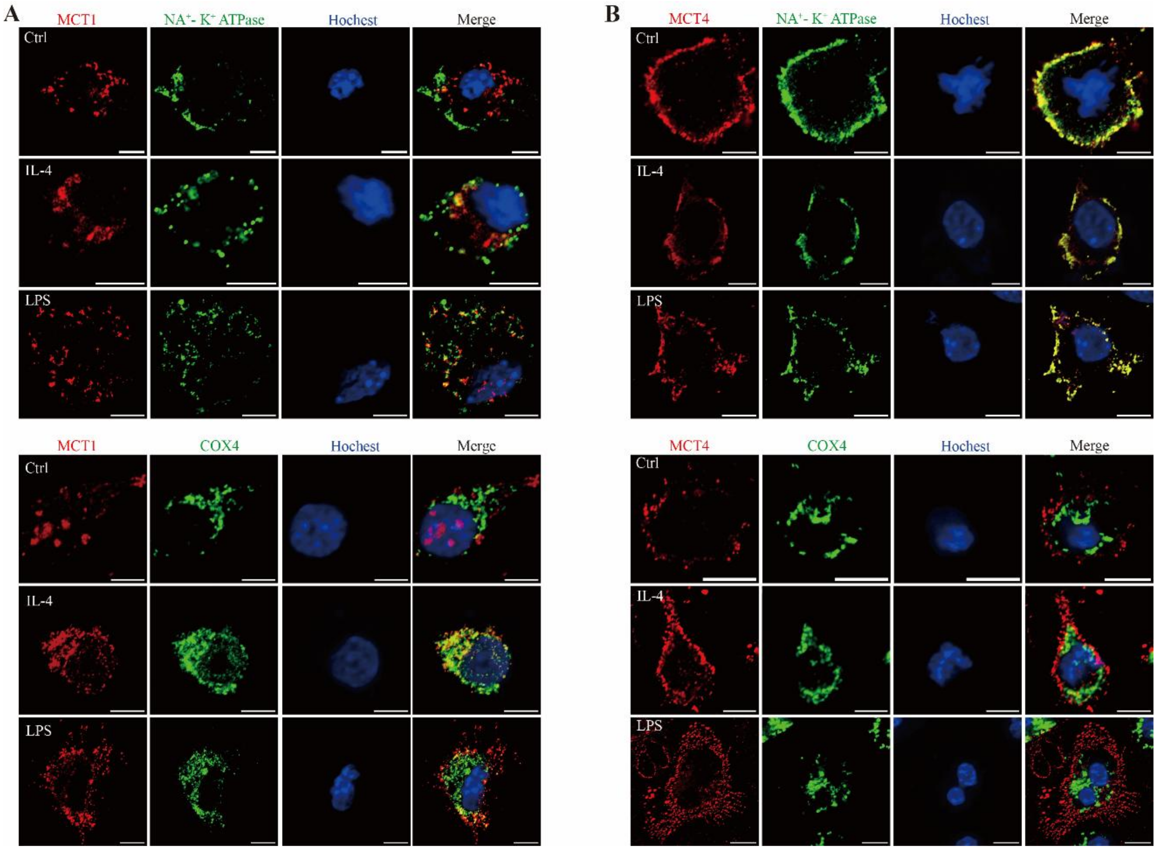

Figure S6. Fluorescent images of Figures 3G-3I with single channel

Scale bar: 5 μm.

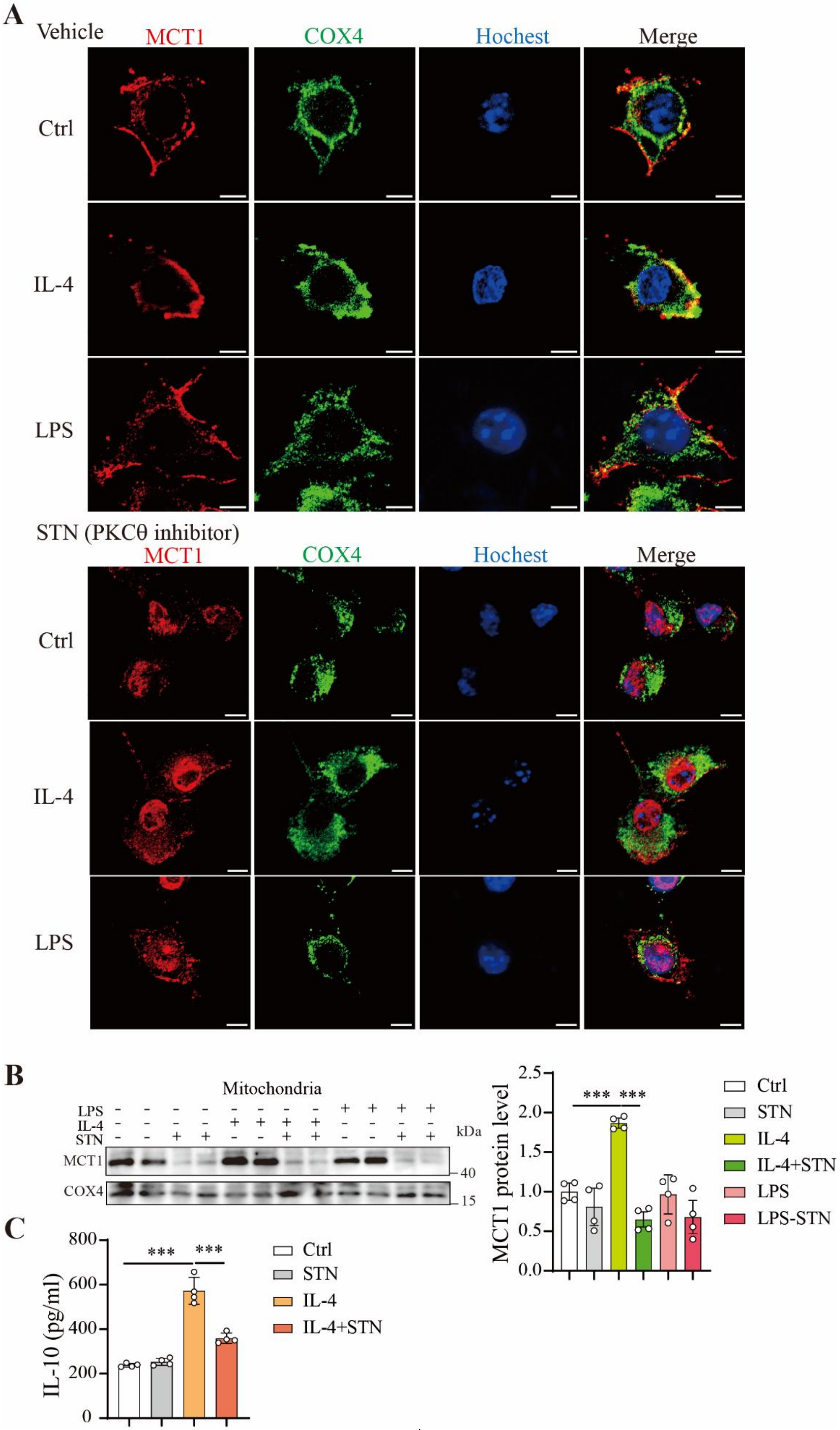
A
Vehicle
MCT1
COX4
Hochest
Merge
Ctrl
IL-4
LPS
STN (PKCθ inhibitor)
MCT1
COX4
Hochest
Merge
Ctrl
IL-4
LPS
B
Mitochondria
LPS
IL-4
STN
kDa
MCT1
COX4
40
15
MCT1 protein level
2.5
2.0
1.5
1.0
0.5
0.0
***
***
Ctrl
STN
IL-4
IL-4+STN
LPS
LPS-STN
C
IL-10 (pg/ml)
800
600
400
200
0
***
***
Ctrl
STN
IL-4
IL-4+STN

Figure S7. PKCθ mediates mitochondrial localization of MCT1 in M2-like macrophages

(A) Representative images of MCT1 and COX4 immunofluorescence staining in BMDMs treated with LPS (100 ng/ml) or IL-4 (50 ng/ml) in the presence or absence of sotrastaurin (STN, PKCθ inhibitor, 100 nM) for 12 h, respectively. Scale bar: 5 μm.

(B) MCT1 protein in isolated mitochondria of BMDM treated with LPS or IL-4 in the presence or absence of STN for 12 h by Western blotting analysis. Quantitative results for MCT1 protein normalized to COX4 are shown on the right, n = 4.

(C) IL-10 secretion of BMDM treated with IL-4 in the presence or absence of STN for 12 h, n = 4. Each point represents as a biological replicate and n represents the number of biological replicates.

Data are presented as mean ± SD. Data are representative of two independent experiments. The *p* values were calculated using one-way ANOVA with Tukey's honest significant difference (HSD) pose hoc analysis. *** $p < 0.001$.

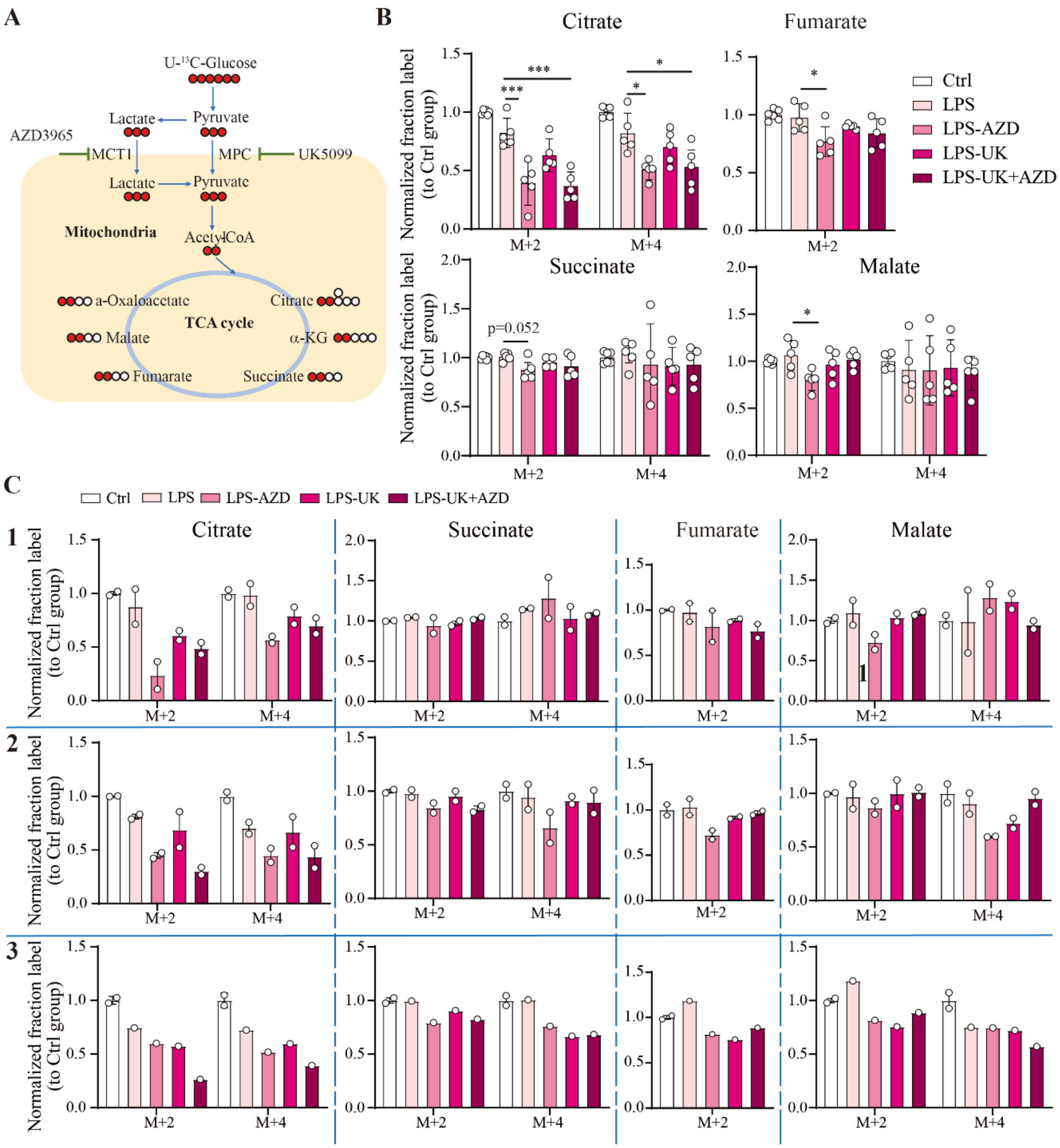

Figure S8. Blocking MCT1 inhibits glucose flux to the TCA cycle in M1-like macrophages

(A) A diagram to depict metabolic flux of U-13C-Glucose.

(B) Normalized fraction label of TCA intermediates after combining results from three independent experiments. BMDMs were treated with LPS (100 ng/mL) for 6 h, followed by incubation for a further 6 h in U-$^{13}C_6$-glucose medium (4.5 g/L) containing LPS, plus 500 nM AZD3965 (AZD), 5 µM UK5099 (UK), or both inhibitors (n = 5 biological repeats). Metabolic flux analysis was performed using isotopologue fractions normalized to the control group.

(C) The individual results of three independent experiments.

Data are presented as mean ± SD. The *p* values were calculated using one-way ANOVA

with Tukey's honest significant difference (HSD) pose hoc analysis (B) o. * $p < 0.05$, ** $p < 0.01$, *** $p < 0.001$.

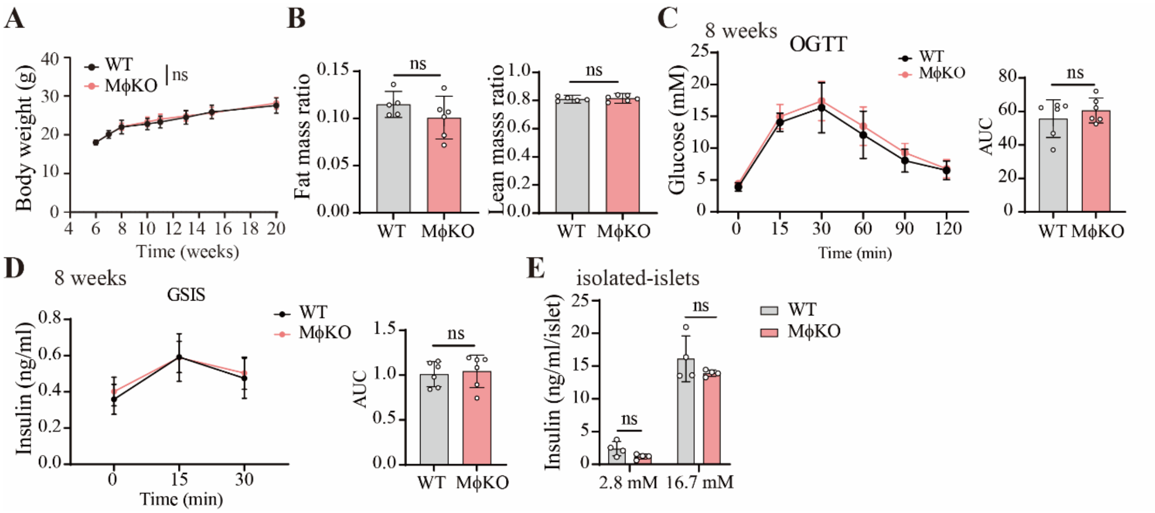

Figure S9. MCT1 loss has no impact on glucose tolerance and islet function in mice fed normal chow

(A) Body weight of WT and MϕKO male mice fed with normal chow, n = 5 for WT mice, n = 6 for MϕKO. (B) Rate of fat mass and lean mass in WT and MϕKO mice at the endpoint in normal chow. (C) Glucose tolerance test (OGTT) of WT and MϕKO mice at the 8th week. The mice were fasted for 16 h before the test, n = 6 mice per group. Quantification of area under curve (AUC) is shown on the right. (D) Glucose stimulated insulin secretion (GSIS) of the mice at the 8th week, n = 6 mice per group. AUC is shown on the right. (E) Glucose stimulated insulin secretion (GSIS) with the isolated islets from mice at the 12th week, n = 4 per group.

Data are presented as mean ± SD. The *p* values were calculated using one-way ANOVA with Tukey's honest significant difference (HSD) pose hoc analysis (A, C and D), unpaired, two-sided Student's t test (B and AUC analysis of D) or two-way ANOVA with Tukey's HSD pose hoc analysis (E). ns for non-significant.

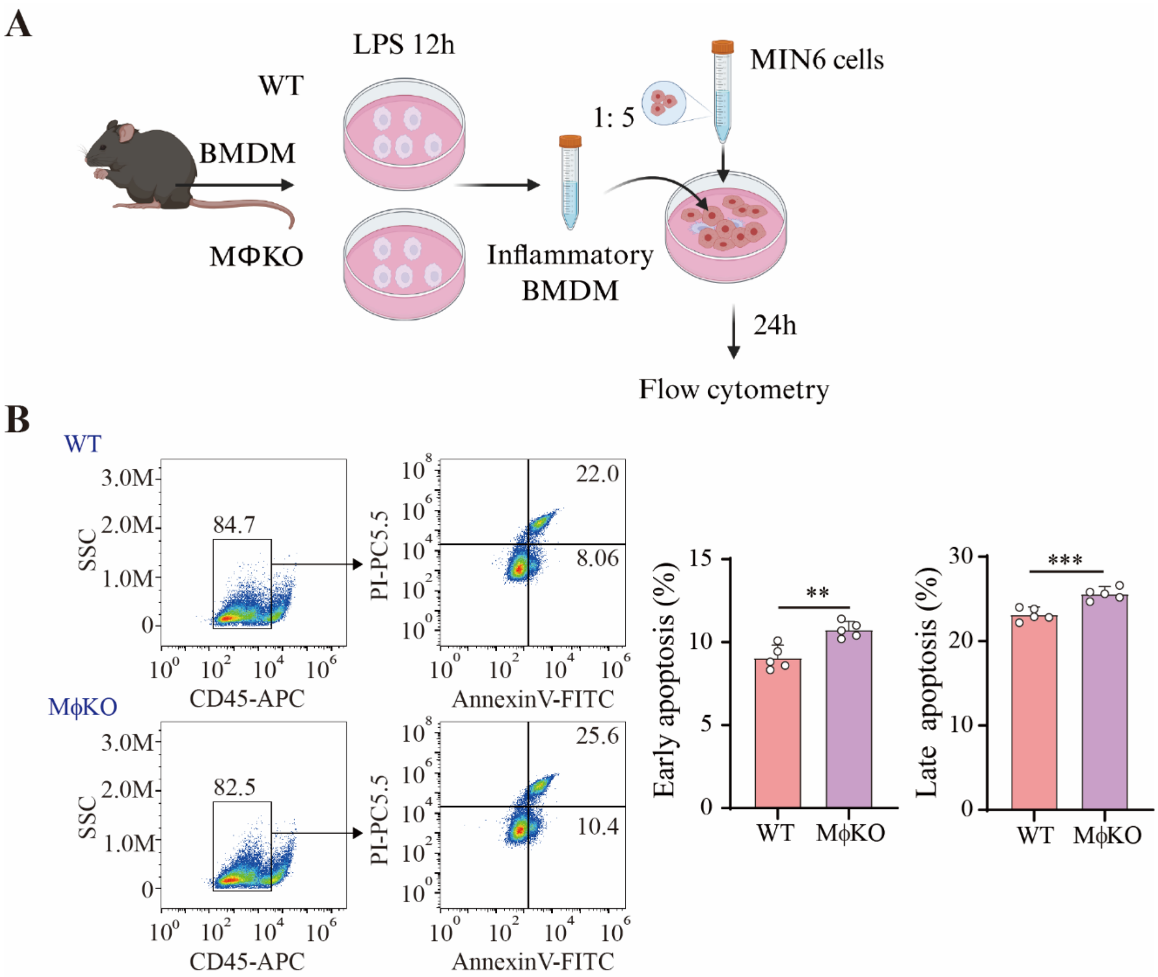

Figure S10. Inflammatory macrophages induce apoptosis of MIN6 cells

(A) Schematic of experimental design for B, created by Biorender.

(B) LPS-treated BMDM were co-cultured with MIN6 cells at a ratio of 1 to 5. Representative flow cytometry plot and ratio of Annexin V^+PI^- early apoptosis cells and Annexin V^+PI^+ late apoptosis cells are shown, n = 5 for each group. Each point represents as a biological replicate and n value represents the number of biological replicates.

Data are presented as mean ± SD. The *p* values were calculated using unpaired, two-sided Student's t test. ** $p < 0.01$, *** $p < 0.001$.

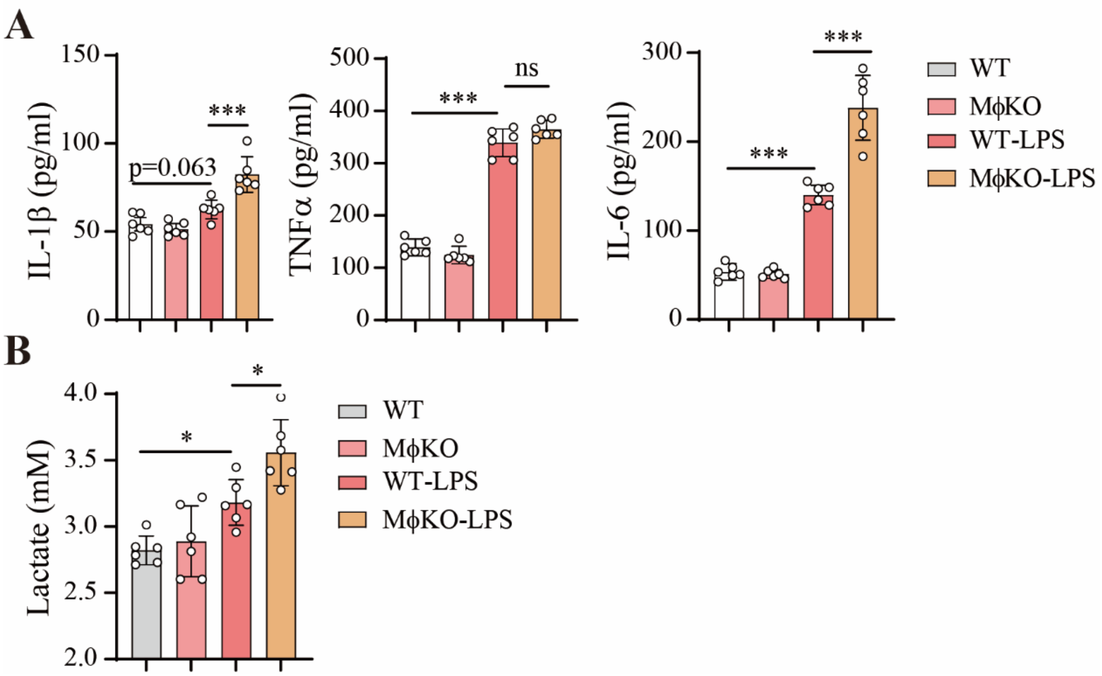

Figure S11. Inflammatory cytokines and lactate concentration for experiment of Figure S10

(A and B) The concentrations of IL-1β, TNFα and IL-6 (A) and lactate (B) in medium when BMDMs were co-cultured with MIN6 cells for 24 h, n = 6. Each point represents as a biological replicate and n value represents the number of biological replicates.

Data are presented as mean ± SD. Data are representative of two independent experiments. The *p* values were calculated using one-way ANOVA with Tukey's honest significant difference (HSD) pose hoc analysis. * $p < 0.05$, ** $p < 0.01$, *** $p < 0.001$. ns for non-significant.

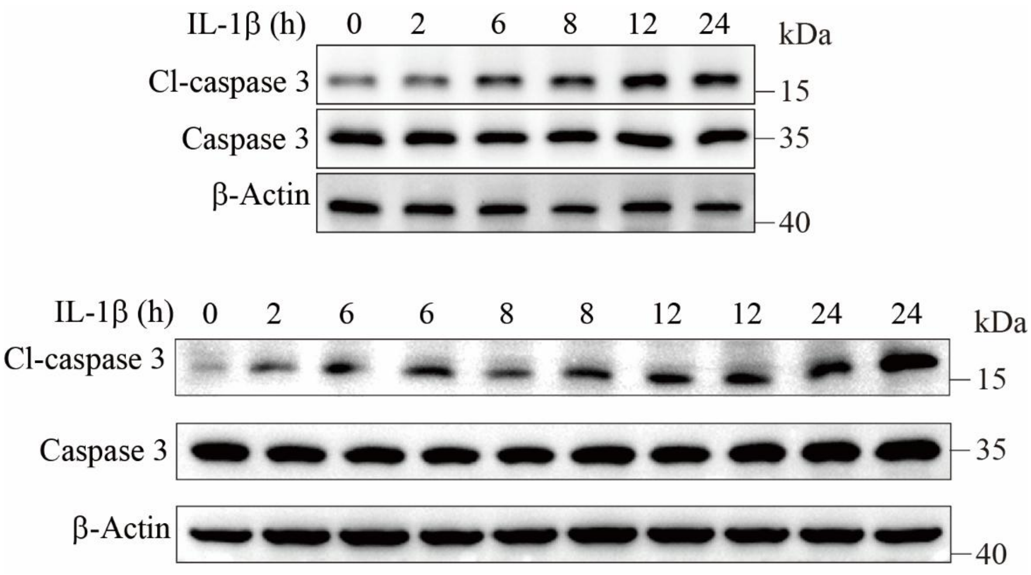

Figure S12. Multiple experiments to show that IL-1β induces apoptosis of MIN6 cells
Western blotting analysis of cleaved-caspase 3 protein level in MIN6 cells, treating with 10 ng/ml IL-1β for different time.
