# Supplementary material for: MCT1‐mediated Lactate Shuttle to Mitochondria Governs Macrophage Polarization and Modulates Glucose Homeostasis by Affecting β Cells
Source: Adv Sci (Weinh). 2025 Jul 14;12(38):e14760. doi: 10.1002/advs.202414760 (PMC12520533; doi:10.1002/advs.202414760)
Supplement: Supplementary file 2 — Supplemental Table 1 [file ADVS-12-e14760-s001.docx]

**MCT1-mediated lactate shuttle to mitochondria governs macrophage polarization and modulates glucose homeostasis by affecting β cells**

*Lingling Chen^1^, Yijun Lin^2^, Xinyu Zhu^3^, Shixuan Zhuo^1^, Zixuan Li^1^, Cheng Guo^1^, Xiaoyi Ye^1^, Jinzhu Chen^1^, Shuying Wang^1^, and Yan Chen^1,3,*^*

**Supplemental Table**

**Table S1. Detailed information of reagents, kits and PCR primer sequence used in this study**

| REAGENT or RESOURCE SOURCE IDENTIFIER | | SOURCE | IDENTIFIER |
| --- | --- | --- | --- |
| **Antibodies** | | | |
| Anti- MCT1/SLC16A1 Rabbit pAb | Abclonal | | Cat# A3013 |
| Anti-MCT1 Polyclonal | Proteintech | | Cat# CL488-20139 |
| Anti-Phospho-(Ser/Thr) PKA Substrate mAb | Cell Signaling Technology | | Cat# 9621S |
| Anti-Caspase3 mAb | Cell Signaling Technology | | Cat# 9662S |
| Anti-Cleaved Caspase 3 (Asp175) | Cell Signaling Technology | | Cat# 9661S |
| Anti-IL-1β (D3U3E) mAb | Cell Signaling Technology | | Cat# 12703S |
| Anti-F4/80 (D4C8V) XP® mAb | Cell Signaling Technology | | Cat# 30325S |
| Anti-Glucagon (ab48287) | Abcam | | Cat# 92517 |
| Anti-β-Actin (2A3) | Santa Cruz Biotechnology | | Cat# 517582 |
| Anti-COX4 (4D11-B3-E8) | Santa Cruz Biotechnology | | Cat# 517553 |
| Anti-NA+/K+ ATPase α-1, clone C464.6 | Sigma-Aldrich | | Cat# 05-369-25UG |
| Anti-SLC16A3 Polyclonal | Thermo Fisher | | Cat# 106683 |
| Anti-human/mouse VDAC1 mAb | Cell Signaling Technology | | Cat# 4661 |
| Anti-LDHA Rabbit | Proteintech | | Cat# 19987-1-AP |
| Anti-LDHB Rabbit | Proteintech | | Cat# 81963-1-RR |
| Anti-MCT4/SLC16A3 Rabbit pAb | Abclonal | | Cat# A10548 |
| Anti-INS Monoclonal | Proteintech | | Cat# 66198-1-IG |
| Anti-GAPDH Monoclonal | Proteintech | | Cat# 60004-1-IG |
| Anti-human/mouse PKCθ mAb | Cell Signaling Technology | | Cat# 13643 |
| **Antibodies: flow cytometry** | | | |
| Fixable viability 510 | BD Biosciences | | Cat# 564406 |
| APC-Cy7 Rat Anti-mouse CD45 | BD Biosciences | | Cat# 561037 |
| CD11b Monoclonal (M1/70), FITC | Invitrogen | | Cat# 11-0112-82 |
| F4/80 Monoclonal , PEeFluor™-610 | Invitrogen | | Cat# 61-4801-82 |

| **Chemicals or reagents** |  |  |
| --- | --- | --- |
| D-(+)-Glucose | Sigma-Aldrich | Cat# G876 |
| L-lactate | Sangon biotech | Cat# 79-33-4 |
| NAOH | Sigma-Aldrich | Cat# 655104 |
| Lipopolysaccharides | MedChemExpress | Cat# HY-D1056 |
| AZD3965 | CSNpharm | Cat# 13789 |
| IL-4/Interleukin-4 | Novoprotein | Cat# CK74 |
| FCCP | MedChemExpress | Cat# 100410 |
| Rotenone | Sigma-Aldrich | Cat# R8875 |
| Antimycin A | Sigma-Aldrich | Cat# A8674 |
| Oligomycin A | MedChemExpress | Cat# 16589 |
| Mouse IL-1β | Novoprotein | Cat# C042 |
| 3-Hydroxybutyric Acid | Sigma-Aldrich | Cat# 52017 |
| 3-Chloro-5-hydroxybenzoic acid | MedChemExpress | Cat# 547339 |
| IBMX | MedChemExpress | Cat# HY-12318 |
| Forskolin | MedChemExpress | Cat# HY-15371 |
| H89 | MedChemExpress | Cat# HY-15979A |
| Sodium oxamate | MedChemExpress | Cat# 565731 |
| BD FACS^TM^Lysing solution | BD Biosciences | Cat# 349202 |
| HBSS | ThermoFisher | Cat# 14175079 |
| VB124 | MedChemExpress | Cat# HY-139665 |
| UK5099 | MedChemExpress | Cat# HY-15475 |
| U-13C6 Glucose | CIL | Cat# CLM-1396-1 |
| Pyruvate sodium | Sigma | Cat# P5280 |
| Collagenase P | Sigma-Aldrich | Cat# 11213857001 |
| **Critical commercial assays** |  |  |
| Lactate Analysis Kit | NJJCBIO | Cat# A019-2-1 |
| Mouse IL-1β ELISA Kit | Mlbio | Cat# 098416 |
| Mouse TNF-a ELISA Kit | Mlbio | Cat# 002095 |
| Mouse IL-6 ELISA Kit | Mlbio | Cat# 098430 |
| Mouse ATP ELISA Kit | Mlbio | Cat# 203922 |
| Mouse cAMP ELISA Kit | Mlbio | Cat# 057902 |
| Mouse IL-1β ELISA Kit | Absin | Cat# abs520001 |
| Mouse TNFα ELISA Kit | Absin | Cat# abs5520033 |
| Mouse IL-6 ELISA Kit | Absin | Cat# abs520004 |
| Mouse IL-10 ELISA Kit | Absin | Cat# abs520005 |
| Human/Mouse TGF-β1 ELISA Kit | Multisciences | Cat# EK981 |
| TUNEL BrightGREEN Apoptosis Kit | Vazyme | Cat# A112-01 |
| High Sensitive Mouse Insulin Kit | Ezassasy | Cat# MS200 |
| Mitochondria Isolation Kit | MedChemExpress | Cat#HY-K1060 100T |
| Apoptosis Detection Kit | BD Biosciences | Cat# 556547 |

| **Experimental models, Cell lines** | | |
| --- | --- | --- |
| HEK293T | | |
| RAW264.7 |  |  |
| MIN6 | | |
| L929 |  |  |
| **Experimental models, Organisms/strains** | | |
| Mouse: C57BL/6J | | |
| Mouse: Slc16a1^f/f^ Lyz2^cre/-^ |  |  |
| **shRNA** | | |
| mus-sh*Slc16a1*-F | CcggccCCAGTGAAGTATCATGGATATCTCGAG ATATCCATGATACTTCACTGGggTTTTTg | |
| mus-sh*Slc16a1*-R | AattcaaaaaccCCAGTGAAGTATCATGGATAT CTCGAGATATCCATGATACTTCACTGGgg | |
| **PCR or Q-PCR primer** | | |
| mus-*Actinb*-F | CTAAGGCCAACCGTGAAAAG | |
| mus-*Actinb*-R | ACCAGAGGCATACAGGGACA | |
| mus-*Slc16a1*-F | ggatatcatctataatgttggctgtc | |
| mus-*Slc16a1*-R | gctgccgtatttattcaccaa | |
| mus-*Slc16a3*-F | gttctggccctcattagctct |  |
| mus-*Slc16a3*-R | ctaggggcagcagagaatgt | |
| mus-*Arg1*-F | GAATCTGCATGGGCAACC |  |
| mus-*Arg1*-R | GAATCCTGGTACATCTGGGAAC | |
| mus-*Cd206*-F | CCACAGCATTGAGGAGTTTG |  |
| mus-*Cd206*-R | ACAGCTCATCATTTGGCTCA | |
| mus-*Ym1*-F | GGTCTGAAAGACAAGAACACTGAG | |
| mus-*Ym1*-R | GAGACCATGGCACTGAACG | |
| mus-*Il1b*-F | AGTTGACGGACCCCAAAAG |  |
| mus-*Il1b*-R | AGCTGGATGCTCTCATCAGG | |
| mus-*Tnfa*-F | CTGTAGCCCACGTCGTAGC |  |
| mus-*Tnfa*-R | TTGAGATCCATGCCGTTG | |
| mus-*Il6*-F | GCTACCAAACTGGATATAATCAGGA | |
| mus-*Il6*-R | CCAGGTAGCTATGGTACTCCAGAA | |
| olMR3066 | CCCAGAAATGCCAGATTACG | |
| olMR3067 | CTTGGGCTGCCAGAATTTCTC | |
| olMR3068 | TTACAGTCGGCCAGGCTGAC | |
| MCT1-P5 | TGTACCAGCCACCGTCCTT | |
| MCT1-P6 | CATCTTGCCTGAGCGTCTAA | |
| mus-*Caspase3*-F | ATGGAGAACAACAAAACCTCAGT | |
| mus-*Caspase3*-R | TTGCTCCCATGTATGGTCTTTAC | |
| mus-*Caspase8*-F | TGCTTGGACTACATCCCACAC | |
| mus-*Caspase8*-R | TGCAGTCTAGGAAGTTGACCA | |
| mus-*Caspase9*-F | TCCTGGTACATCGAGACCTTG | |
| mus-*Caspase9*-R | AAGTCCCTTTCGCAGAAACAG | |
| **Recombinant DNA** |  | |
| pLVX-Fila-Cyto |  |  |
| pLVX-Fila-Mito |  |  |
| PLKO.1 |  |  |
| **Software and algorithms** |  |  |
| GraphPad Prism 9 |  |  |
| ImageJ |  |  |
| ZEN |  |  |
| FV10-ASW 4.2 |  |  |
| QuantStudio 6 Real -Time PCR System | Thermo Fisher |  |
| LSM880NLO FLIM | Carl Zeiss |  |
| XF24/XF96 | Seahorse |  |
| CytoFlex LX | Beckman Coulter |  |
| Flow jo 10 | | |
| **Others** | | |
| RPIM 1640 | GIBCO |  |
| DMEM | GIBCO |  |
| RPIM 1640 (-Glucose) | GIBCO |  |
| Dialysis fetal bovine serum (DFBS) |  |  |
| Seahorse XFp Media & Calibrant |  |  |
